# Mapping Cognitive Oncology: A Decade of Trends and Research Fronts

**DOI:** 10.3390/medsci13030191

**Published:** 2025-09-15

**Authors:** Anna Tsiakiri, Akyllina Despoti, Panagiota Koutsimani, Kalliopi Megari, Spyridon Plakias, Angeliki Tsapanou

**Affiliations:** 1 Department of Neurology, Democritus University of Thrace, 68100 Alexandroupolis, Greece; 2 Hellenic Neuropsychological Society, 11636 Athens, Greece; a.despoti@yahoo.com (A.D.); pikoutsi@psy.auth.gr (P.K.); kmegari@psy.auth.gr (K.M.); tsapanou@upatras.gr (A.T.); 3 Clinical Ergospirometry, Exercise & Rehabilitation Lab, School of Medicine, National and Kapodistrian University of Athens, 11527 Athens, Greece; 4 Brain and Performance Center, Dubai, United Arab Emirates; 5 School of Social Sciences and Humanities, Department of Psychology, University of Western Macedonia, 53100 Florina, Greece; 6 Lab of Neuropsychology & Behavioral Neuroscience, School of Psychology, Aristotle University of Thessaloniki, 54124 Thessaloniki, Greece; 7 City College, Europe Campus, University of York, 54626 Thessaloniki, Greece; 8 Department of Physical Education and Sport Science, University of Thessaly, 42100 Trikala, Greece; spyros_plakias@yahoo.gr; 9 Department of Speech and Language Therapy, School of Health Rehabilitation, University of Patras, 26504 Patras, Greece

**Keywords:** cancer-related cognitive impairment, neuropsychology, bibliometric analysis, science mapping, cognitive rehabilitation

## Abstract

Background: Cognitive and neuropsychological effects of cancer and its treatments have gained increasing attention over the past decade, with growing evidence of persistent deficits across multiple cancer types. While numerous studies have examined these effects, the literature remains fragmented, and no comprehensive bibliometric synthesis has been conducted to map the field’s intellectual structure and emerging trends. Methods: A bibliometric and science mapping analysis was performed using the Scopus database to identify peer-reviewed articles published between 2015 and 2025 on neuropsychological or cognitive outcomes in adult cancer populations. Data from 179 eligible publications were analyzed with VOSviewer and Microsoft Power BI, applying performance metrics and network mapping techniques, including co-authorship, bibliographic coupling, co-citation, and keyword co-occurrence analyses. Results: Publication output increased steadily over the decade, with leading contributions from the Journal of Neuro-Oncology, Psycho-Oncology, and Brain Imaging and Behavior. Co-citation analysis identified three core intellectual pillars: (i) clinical characterization of cancer-related cognitive impairment, (ii) mechanistic and neuroimaging-based investigations, and (iii) neurosurgical and neuropathological research in brain tumors. Keyword mapping revealed emerging themes in sleep and circadian rhythm research, biological contributors to cognitive decline, and scalable rehabilitation strategies such as web-based cognitive training. Collaborative networks, while showing dense local clusters, remained moderately fragmented across disciplines. Conclusions: This review provides the first quantitative, decade-spanning map of cognitive oncology research, highlighting both consolidated knowledge areas and underexplored domains. Future efforts should prioritize methodological standardization, cross-disciplinary collaboration, and integration of cognitive endpoints into survivorship care, with the ultimate aim of improving functional outcomes and quality of life for cancer survivors.

## 1. Introduction

Recent studies have shown that the nervous system plays an essential role in the development and spread of cancer [1]. Cognitive dysfunction is a frequent and clinically relevant consequence of cancer treatment, particularly following chemotherapy. Breast cancer patients, for example, often perform worse on cognitive tests even six months after completing chemotherapy, showing greater impairment than patients with other types of cancer. These findings suggest that chemotherapy may contribute to persistent cognitive decline, with significant implications for rehabilitation strategies aimed at improving cognitive functioning and everyday independence. Language dysfunction is also a critical issue in patients with brain tumors, affecting quality of life and daily activities [2]. Moreover, chemotherapy-treated breast cancer patients have demonstrated poorer performance on measures of Instrumental Activities of Daily Living (IADL), reflecting greater difficulty with complex daily tasks. In contrast, the IADL performance of the other three study groups, those not receiving chemotherapy (e.g., patients undergoing radiation, hormone therapy, or surgery), remained unchanged. Importantly, a composite neuropsychological performance score based on cognitive assessments in domains such as memory, executive functioning, and attention was found to be a significant predictor of IADL performance, with cognitive ability declining as disability severity increased [3,4].

However, cognitive difficulties are not limited to patients undergoing chemotherapy. Several studies have reported that signs of cognitive dysfunction may appear even before the initiation of cancer treatment, or in patients not receiving pharmacological interventions. Cognitive deficits have also been documented following other cancer therapies such as radiation, hormone therapy, or surgery, and are present even in non-central nervous system cancers. These deficits can significantly affect brain functioning and quality of life. Given the complex and multifactorial nature of these impairments, some researchers argue that the term “cancer-related cognitive impairment (CRCI)” is more accurate than general descriptors such as “cognitive dysfunction” or the colloquial “chemobrain.” The term CRCI better captures the range, timing, and mechanisms of cognitive changes observed across different cancer types and treatment stages [5]. These studies’ findings have long-term implications for clinical care and patient quality of life. They emphasize the critical role that the type of surgery plays in the development of postoperative cognitive dysfunction (POCD). From a clinician’s perspective, it is important for health policy professionals to understand that patients with mild POCD may experience persistent symptoms over an extended period [6].

Bibliometric analysis has emerged as a robust method for evaluating the structure, development, and dynamics of research fields by systematically mapping the scientific literature through quantitative techniques [7]. Unlike traditional reviews, which often synthesize findings within a narrower scope, bibliometric analysis enables the identification of large-scale patterns such as authorship networks, journal productivity, and thematic trends, thus providing a comprehensive overview of a field’s intellectual landscape [8]. This method is particularly valuable in rapidly expanding or interdisciplinary areas where the volume of publications makes manual synthesis impractical. For example, recent bibliometric studies have examined stroke neurorehabilitation [9,10], artificial intelligence in diagnosis [11], and youth physical education trends [12]. These studies not only highlight the dominant contributors and outlets in each field but also uncover emerging research fronts, knowledge gaps, and methodological shifts. In this context, bibliometric analysis serves as a powerful tool for guiding evidence-based research strategies and fostering targeted scientific collaboration.

Despite the increasing volume of publications, the cognitive oncology literature remains fragmented across subfields and disciplines, and no comprehensive bibliometric synthesis has yet been conducted to map its development. Existing reviews typically focus on narrow clinical topics or specific treatment modalities, overlooking the broader intellectual and collaborative landscape. The present study aims to address this gap by conducting a bibliometric and science mapping analysis of research related to neuropsychological and cognitive effects of cancer. By analyzing patterns of authorship, publication sources, co-citation networks, and thematic clusters of keywords, we seek to uncover the structural and conceptual organization of the field. This approach provides a quantitative overview of influential contributors, dominant topics, and emerging research directions. Ultimately, this bibliometric review intends to serve as a foundational resource for researchers, clinicians, and stakeholders interested in understanding the evolution and future trajectory of cognitive oncology as a field of inquiry.

## 2. Methods

### 2.1. Search

The search for relevant literature was conducted on 22 May 2025, using the Scopus database. The search targeted the title, abstract, and keyword fields, focusing on the identification of research articles examining neuropsychological aspects related to cancer. To maintain a comprehensive scope, the search was limited only by publication year and language, excluding non-article documents. The Boolean search string was designed to include articles referring to neuropsychological outcomes or cognitive impairments in the context of cancer. The final expression used was TITLE-ABS-KEY ((“Neuropsychological exponents” OR “cognitive deficits” OR “cognitive disorders”) AND cancer) AND PUBYEAR > 2014 AND PUBYEAR < 2026 AND (LIMIT-TO (DOCTYPE, “ar”)) AND (LIMIT-TO (LANGUAGE, “English”)). This search yielded peer-reviewed articles published between 2015 and 2025, written in English, ensuring the inclusion of the most recent and relevant empirical evidence in the field.

### 2.2. Selection Criteria

Articles retrieved from the initial search were screened for eligibility based on specific inclusion and exclusion criteria. The selection focused on peer-reviewed journal articles written in English and published between 2015 and 2025. Eligible articles addressed neuropsychological outcomes or cognitive deficits in the context of cancer, regardless of article type (e.g., original research, review, or theoretical studies) (Table 1).

Studies were excluded if they involved non-human subjects (e.g., rats), presented as case reports, focused exclusively on pediatric populations, or were protocol articles. Pediatric populations were excluded due to fundamental developmental differences in neurocognitive functioning and treatment response, which limit comparability with adult data. Furthermore, articles written in languages other than English, or published in non-academic outlets such as magazines, conference proceedings, or reports, were not considered.

Initial screening was based on title and abstract content. When necessary, full-text access was used to verify eligibility, provided the article was accessible. Although the Boolean expression included limits based on document type, all records were individually screened to ensure alignment with the research scope. Since this study is a bibliometric review rather than a systematic one, formal tools like PRISMA or AMSTAR were not applied.

### 2.3. Data Extraction and Software

Bibliographic data for all included articles were extracted from the Scopus database in the form of a CSV file. This file contained comprehensive metadata for each publication, including authorship, title, keywords, journal, year of publication, citations, and abstracts, with the exception of funding information which was not included in the export format. The CSV file was subsequently imported into the open-source software VOSviewer (version 1.6.20.0) [13,14] for the purposes of bibliometric mapping and co-occurrence analysis. To complement this, additional visualizations such as frequency charts and distribution graphs were generated using Microsoft Power BI (Office 365, Microsoft Corporation, Redmond, WA, USA) [15]. The combination of both tools allowed for multidimensional data representation and exploration.

### 2.4. Bibliometric Analysis

The bibliometric analysis conducted in this study integrated both performance analysis and science mapping techniques, based on established practices in the field [16,17]. The analyses were carried out using VOSviewer (version 1.6.20.0), supported by preprocessing procedures including the creation of thesaurus files to correct inconsistencies in author names, journal titles, and keywords. The performance analysis aimed to evaluate publication productivity in terms of authorship and source contribution. Specifically, authors were included in the analysis if they had contributed at least four documents, and the weight attributed to each node in the network visualization was based on the total number of documents. Similarly, for the analysis of sources, a minimum threshold of three documents per source was applied, with weights also determined by document count. These data were presented through summary tables and graphical outputs, providing an overview of the most prolific contributors in the field.

In terms of science mapping, four distinct techniques were applied to explore the structural and collaborative patterns within the literature: co-authorship analysis, bibliographic coupling, co-citation analysis, and keyword co-occurrence analysis. In the co-authorship analysis, the unit of analysis was institutional affiliation, with a minimum threshold of two documents required for inclusion. The visual output was a network diagram, with node size based on the number of documents per organization. For the bibliographic coupling analysis, the unit of analysis was the journal or source, again requiring a minimum of two documents. The analysis revealed thematic overlap among sources by identifying shared references, and was also represented through a network visualization weighted by document count. The co-citation analysis focused on authors, assessing intellectual linkages based on how frequently authors were cited together in other works [8,16]. A threshold of twenty citations was applied to ensure clarity and significance in the resulting network, which was weighted by citation count. Lastly, the co-occurrence analysis utilized author keywords as the unit of analysis, examining how often specific keywords appeared together across publications. A minimum occurrence of three keywords was set, with the resulting network diagram weighted by keyword frequency. All network visualizations were automatically clustered by VOSviewer, based on link strength and spatial proximity [10,18,19]. Table 2 summarizes the methodological framework applied to each science mapping technique, including units of analysis, minimum thresholds, weight metrics, and type of visualization employed:

## 3. Results

### 3.1. Included Documents

**Figure 1:**
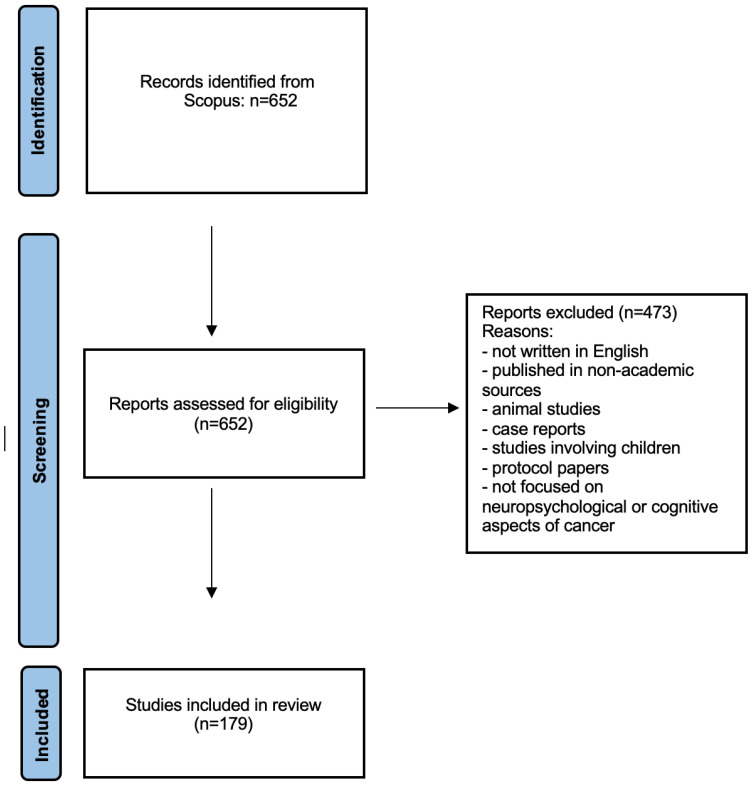
Article selection process.

As illustrated in the flow diagram in Figure 1, the initial search conducted in the Scopus database yielded a total of 652 documents. Following the application of the predefined inclusion and exclusion criteria, 179 articles were retained for analysis and are listed in the Supplementary File accompanying this study. The remaining 473 documents were excluded for one or more of the following reasons: duplicate entries, documents not written in English, articles published in non-peer-reviewed or non-academic sources, studies involving non-human subjects (e.g., rats), case reports, pediatric populations, protocol papers, or documents that did not sufficiently focus on neuropsychological or cognitive aspects related to cancer. Each article was screened based on its title and abstract, and full texts were consulted where necessary and accessible. This selection process ensured the inclusion of articles most relevant to the objectives of this bibliometric study, providing a reliable foundation for the subsequent performance and science mapping analyses. All studies are presented in Table S1.

### 3.2. Bibliometric Performance Analysis

To assess the productivity and impact of individual contributors within the field, a performance analysis was conducted focusing on the number of publications and associated citations for each author. As shown in Table 3, the most prolific authors within the final sample of 179 articles were Francis Eustache, Bénédicte Giffard, and Florence Joly, each contributing five documents. These authors are internationally recognized for their research in neuropsychology, with a particular emphasis on cognitive impairment and rehabilitation in oncological populations. Notably, Margriet M. Sitskoorn, although represented with four publications, accumulated the highest number of citations (*n* = 191), indicating the substantial academic influence and visibility of her work. This distribution suggests a relatively concentrated core of researchers consistently contributing to the literature on neurocognitive outcomes in cancer, with a broader periphery of authors contributing more sporadically. The predominance of specific individuals in both productivity and citation count reflects both sustained engagement in the topic and recognition by the wider research community.

In addition to author productivity, a source-level performance analysis was performed to identify the most active and influential journals within the dataset. As presented in Table 4, the Journal of Neuro-Oncology was the most frequent outlet, with nine included articles and a total of 194 citations, highlighting its centrality in disseminating research on cognitive sequelae in neuro-oncological contexts. Other journals with high publication volume included Psycho-Oncology (6 articles, 99 citations) and Brain Imaging and Behavior (5 articles, 132 citations), both of which emphasize interdisciplinary approaches combining oncology, neuroimaging, and cognitive sciences. Interestingly, the journal Neuro-Oncology, although contributing only four documents, exhibited the highest citation count overall (*n* = 234), suggesting that its publications in this field are of particularly high impact. Similarly, Oncologist demonstrated a strong citation-to-publication ratio, further underscoring the importance of citation-based metrics in evaluating scholarly influence beyond raw publication counts.

### 3.3. Science Mapping

#### 3.3.1. Co-Authorship Analysis

**Figure 2:**
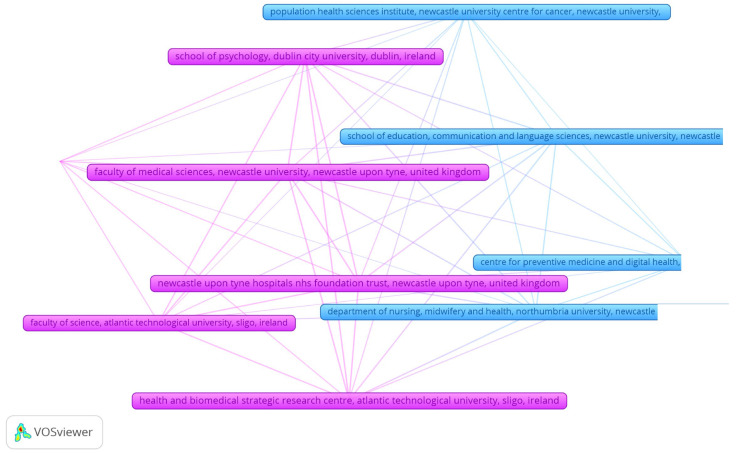
Co-authorship Network Based on Organizational Affiliation. Each node represents an institutional affiliation, and the size of the node corresponds to the number of documents associated with that institution. Connections (edges) indicate co-authorship ties, reflecting collaborative efforts across institutions. The visualization was generated using VOSviewer with a minimum threshold of two documents per organization. Clusters are color-coded based on co-authorship proximity and were automatically assigned by the software using modularity-based clustering.

To explore collaborative patterns at the institutional level, a co-authorship analysis was conducted using organizations as the unit of analysis. As illustrated in Figure 2, the network comprises organizations that have co-authored at least two documents within the dataset. Each node represents a unique organization, and the links between nodes indicate co-authored publications, thereby reflecting inter-institutional research collaborations. The size of each node is proportional to the number of documents affiliated with that organization, while node color reflects clusters identified through modularity-based clustering using VOSviewer, signifying groups of institutions with closer co-authorship ties. The resulting map reveals a moderately fragmented network, with some densely connected clusters and a number of weaker peripheral connections. Two main clusters emerge: one predominantly composed of institutions in clinical and biomedical research (e.g., Faculty of Medical Sciences, Newcastle, Newcastle upon Tyne Hospitals, Health and Biomedical Strategi, and School of Psychology, Dublin C), and another centered around public health and nursing research (e.g., Population Health Sciences Institute, Department of Nursing, Midwife, and School of Education, Communica).

Of particular interest is the Centre for Preventive Medicine, which appears in both clusters and acts as a bridging node across disciplinary and institutional boundaries, suggesting a potential role in facilitating interdisciplinary collaboration. Despite the presence of these central nodes, the overall structure suggests that institutional collaborations are still emerging, with many partnerships localized within clusters rather than spanning across them. This pattern may indicate either a developmental stage of the research field or disciplinary segmentation, where inter-organizational ties have not yet fully matured across thematic areas.

#### 3.3.2. Bibliographic Coupling Analysis

**Figure 3:**
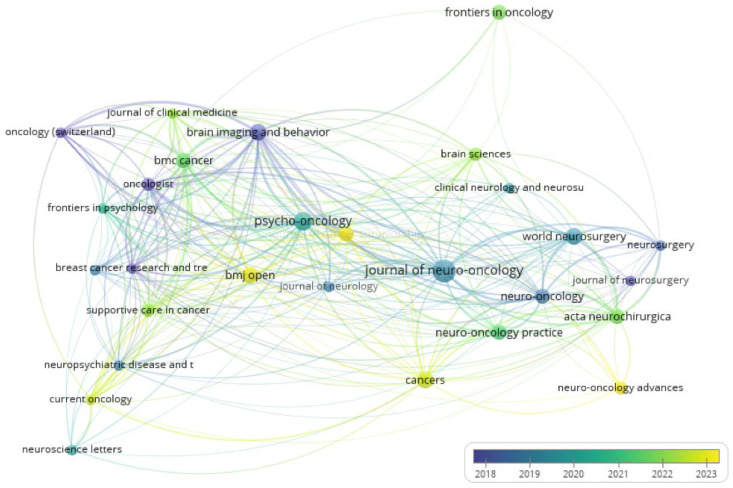
Overlay visualization of bibliographic coupling among sources included in the bibliometric analysis. Each node represents a journal, and its size corresponds to the number of documents published in the dataset. The spatial positioning reflects the strength of bibliographic coupling based on shared references, while the color gradient indicates the average publication year (blue = older; yellow = more recent). A minimum threshold of two documents per source was applied. Visualization generated in VOSviewer using overlay mapping.

To assess the intellectual proximity among publication outlets in the field, a bibliographic coupling analysis was conducted using sources as the unit of analysis. This method identifies journals that share overlapping references, thereby revealing structural and thematic alignments within the field. A minimum threshold of two documents per source was set, and weights were assigned based on the number of documents attributed to each journal. The results are illustrated in the overlay visualization presented in Figure 3. Overlay visualization integrates temporal metadata by displaying the average publication year associated with each source. In this visualization, the position of each node reflects the strength of bibliographic coupling—how many shared references it has with other sources—while the color gradient represents the average publication year, ranging from cooler colors (blue) for older sources to warmer tones (yellow) for more recent ones. This format offers a dynamic perspective by showing not only the structural role of each source but also its temporal contribution to the literature.

As shown, the Journal of Neuro-Oncology occupies a highly central position, with extensive bibliographic linkages, though shaded in cooler hues, suggesting that its influence is largely based on earlier publications in the timeframe. Similarly, Brain Imaging and Behavior appears in a central position but with a darker blue color, indicating that its contributions are rooted more in the earlier years of the decade. In contrast, journals such as BMJ Open and Cancers are both positioned centrally and shaded in yellow, reflecting very recent publishing activity and strong engagement with the emerging literature. Psycho-Oncology is also well-connected and shaded in green–yellow tones, indicating sustained and relatively recent contributions. More peripheral journals, such as Neuro-Oncology advances and Current Oncology contribute with fewer shared references, but their warmer hues suggest that they represent newer areas of activity and thematic expansion within cognitive oncology research. Overall, this pattern highlights a dual trend: foundational contributions from journals like Journal of Neuro-Oncology and Brain Imaging and Behavior established earlier in the decade, complemented by more recent and expanding contributions from outlets such as BMJ Open, Acta Neurochirurgica, and Neuro-Oncology Advances.

#### 3.3.3. Co-Citation Analysis

**Figure 4:**
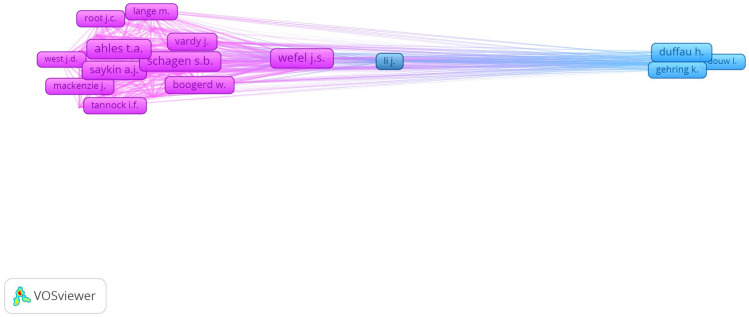
Network visualization of author co-citation relationships based on the documents included in the analysis. Each node represents an author, and the size of the node reflects the number of times the author has been co-cited (i.e., cited together with another author in the same documents). Edges between nodes indicate co-citation links, with thicker lines representing stronger co-citation frequencies. The minimum threshold for inclusion was set at 30 citations, and clustering was performed automatically by VOSviewer, resulting in color-coded groups that reflect distinct research subfields or intellectual communities.

To examine the intellectual structure and key contributors within the field, a co-citation analysis was conducted using authors as the unit of analysis. Co-citation analysis identifies how frequently pairs of authors are cited together across the literature, revealing clusters of scholars whose work is conceptually or thematically aligned. In this study, a minimum threshold of 30 co-citations was applied to ensure analytical clarity and relevance. The resulting network, illustrated in Figure 4, reveals several densely interconnected clusters, each representing a group of authors who are frequently cited in tandem and therefore form distinct intellectual communities. The most prominent cluster (red) is centered around Wefel J.S., Ahles T.A., and Schagen S.B., whose work predominantly focuses on cognitive dysfunction associated with cancer and chemotherapy (“chemo brain”). This cluster reflects a significant body of literature addressing neuropsychological outcomes in cancer survivors, with strong internal connectivity and numerous cross-links to adjacent clusters. A second cluster (blue) includes author such as Li J., reflecting a research stream emphasizing biological mechanisms, neuroinflammation, and neuroimaging studies. The third cluster (green), anchored by Duffau H., and Aaronson N.K., is oriented more towards neurosurgical approaches, brain tumor pathology, and health-related quality of life, indicating a complementary but distinct thematic focus within the broader field.

#### 3.3.4. Co-Occurrence Analysis

**Figure 5:**
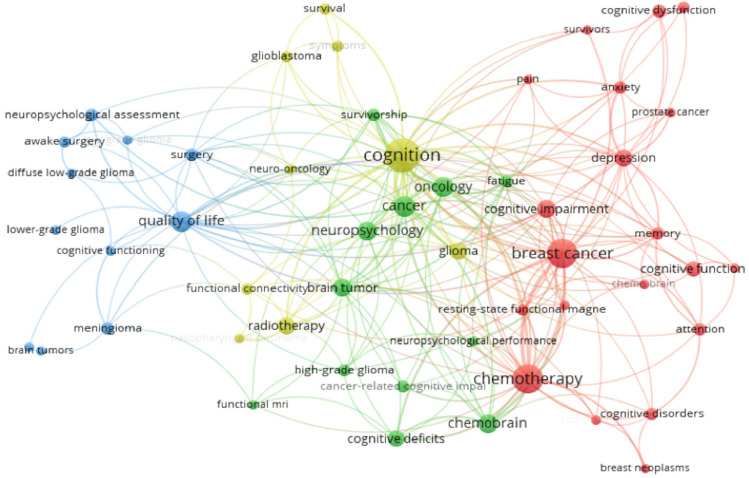
Network visualization of co-occurrence patterns among author keywords appearing in the included documents. Each node represents a keyword, and the size of the node corresponds to the frequency of its occurrence. Links between nodes indicate the number of documents in which the two keywords appear together. Colors represent clusters of thematically related terms, automatically generated by VOSviewer through modularity-based clustering.

To investigate the conceptual landscape of research on neuropsychological and cognitive outcomes in oncology, a co-occurrence analysis of author keywords was conducted. This approach reveals how frequently specific terms appear together in the same documents, thereby highlighting thematic relationships and dominant areas of focus within the literature. A minimum threshold of three keyword occurrences was applied to ensure the inclusion of relevant and recurrent terms. As shown in Figure 5, the resulting network comprises several distinct clusters, each corresponding to a thematic area within the field. The yellow cluster, centered on cognition, includes terms such as glioblastoma, radiotherapy, neuro-oncology, glioma, and functional connectivity. This cluster reflects research focusing on intensive oncological treatments, biological mechanisms, and network-level brain changes associated with cognitive outcomes. The green cluster is anchored by neuropsychology and contains terms like brain tumor, cancer, oncology, chemo brain, and cognitive deficits. It represents the conceptual and methodological core of the field, emphasizing cancer-related cognitive impairment as a recognized neuropsychological outcome across tumor types and treatment modalities. The blue cluster brings together quality of life, surgery, and meningioma, highlighting the literature on neurosurgical approaches, tumor classification, and the balance between treatment efficacy and preservation of cognitive functioning and daily well-being. Finally, the red cluster is oriented toward psychosocial and chemotherapy-related dimensions, comprising terms such as breast cancer, chemotherapy, cognitive dysfunction, depression, anxiety, and memory. This cluster emphasizes the psychological and emotional burden of cancer-related cognitive impairment, particularly in breast cancer survivors. Together, these clusters provide a comprehensive map of the field, spanning mechanistic and treatment-related investigations, core neuropsychological constructs, surgical outcomes, and psychosocial aspects of cancer-related cognitive impairment.

**Figure 6:**
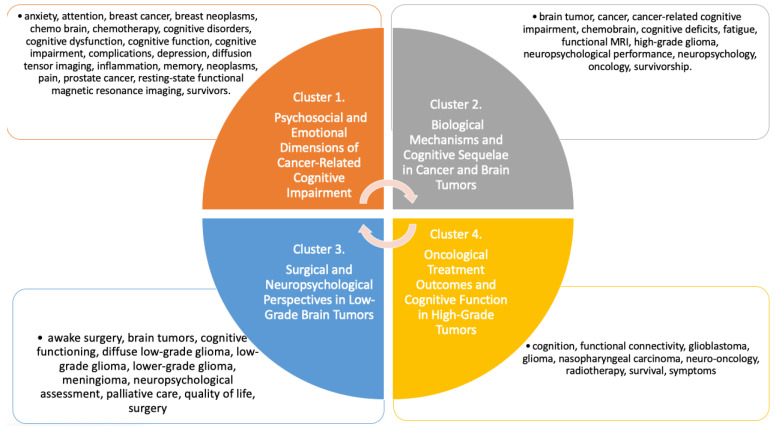
Visualization of the four thematic clusters identified through co-occurrence analysis of author keywords in the included literature. Each cluster represents a distinct conceptual domain within the field of cognitive and neuropsychological outcomes in cancer. The clusters were generated using VOSviewer’s modularity-based clustering algorithm and are labeled based on the dominant themes of the terms they contain.

From the co-occurrence analysis of author keywords in the included publications on neuropsychological and cognitive outcomes in cancer, four distinct clusters were identified. Only keywords that appeared at least three times were considered in the analysis. The clusters, visualized in Figure 6, were generated using VOSviewer’s co-occurrence algorithm, which detects natural groupings based on term frequency and co-occurrence strength within the dataset. Each cluster reflects thematically cohesive areas within the field, as determined algorithmically and visualized by modularity-based color coding.

Cluster 1, titled Psychosocial and Emotional Dimensions of Cancer-Related Cognitive Impairment, includes terms primarily associated with emotional, behavioral, and subjective cognitive difficulties often reported by cancer patients and survivors. Keywords such as anxiety, depression, cognitive dysfunction, pain, and chemo brain indicate a focus on the experiential and symptomatic burden of cancer-related cognitive disturbances, particularly in populations such as breast cancer and prostate cancer patients. This cluster emphasizes the intersection of mental health and cognition within oncological care [20,21,22,23,24,25,26,27,28,29,30,31].

Cluster 2, labeled Biological Mechanisms and Cognitive Sequelae in Cancer and Brain Tumors, includes terms that reflect the biological underpinnings and measurable outcomes of cognitive impairment, such as brain tumor, chemobrain, cognitive deficits, and functional MRI. This cluster focuses on neurobiological assessment and the systemic effects of cancer and its treatments on the brain, with additional emphasis on high-grade gliomas and their neuropsychological consequences [32,33,34,35,36,37,38,39,40,41,42,43,44,45,46,47,48,49,50,51,52,53,54,55,56].

Cluster 3, referred to as Surgical and Neuropsychological Perspectives in Low-Grade Brain Tumors, encompasses terms related to surgical intervention, tumor classification, and neurocognitive evaluation. Keywords such as awake surgery, meningioma, diffuse low-grade glioma, and neuropsychological assessment highlight this cluster’s orientation toward treatment planning and outcome monitoring in patients with slow-growing brain tumors, often within the context of quality-of-life preservation and palliative care [57,58,59,60,61,62,63,64,65,66,67,68,69,70,71].

Cluster 4, titled Oncological Treatment Outcomes and Cognitive Function in High-Grade Tumors, groups terms centered around cognition, radiotherapy, neuro-oncology, and glioblastoma, indicating a thematic focus on intensive oncological treatment and its effects on brain function and survival. Terms such as functional connectivity and symptoms point to a broader consideration of clinical and functional outcomes in aggressive tumor contexts, including nasopharyngeal carcinoma.

Together, these clusters provide a comprehensive overview of the research landscape, revealing how cognitive and neuropsychological dimensions in cancer care are thematically structured across emotional, biological, surgical, and therapeutic domains [72,73,74,75,76,77,78,79,80,81,82,83,84,85,86,87,88,89,90,91,92].

## 4. Discussion

### 4.1. Summary of Key Findings

This bibliometric and science mapping analysis provides the first comprehensive overview of the neuropsychological and cognitive research landscape in oncology from 2015 to 2025. The findings indicate a steadily growing field, with a marked increase in publications after 2020, highlighting the rising academic and clinical attention toward the cognitive and psychological consequences of cancer and its treatment. While data collection extended only until May 2025, the number of studies already published suggests sustained momentum and continued relevance. Key journals such as Journal of Neuro-Oncology, and Brain Imaging and Behavior emerged as central publication platforms, illustrating the interdisciplinary scope of the field—encompassing neuroimaging, behavioral science, oncology, and psychosocial research.

A dense author core was identified, led primarily by Europe-based researchers. Notably, Francis Eustache, Bénédicte Giffard, and Florence Joly (Caen-Normandy University) were among the most prolific contributors, while Margriet M. Sitskoorn (Tilburg University) was the most cited. Despite the presence of high-impact individuals, collaboration at the institutional level appeared limited, with research networks remaining relatively fragmented. Nevertheless, emerging connections—such as between the Faculty of Medical Sciences and the Department of Medicine at Chang Gung School—may signal early developments toward broader cooperation.

Keyword co-occurrence and co-citation analyses revealed four main thematic directions, ranging from psychosocial and emotional aspects of cancer-related cognitive impairment to biological mechanisms, surgical outcomes, and treatment-induced neurotoxicity. These themes illustrate the multifactorial nature of the field and the complementary methodologies applied across studies.

Overall, this analysis highlights key challenges such as limited cross-institutional collaboration, as reflected in the fragmented co-authorship networks. At the same time, it points to promising conceptual integration, particularly through the convergence of neuroimaging, cognitive science, and patient-centered outcomes. These findings offer a clearer conceptual map of cognitive oncology and support the development of a more cohesive, interdisciplinary, and patient-focused research agenda.

### 4.2. Interpretation of Thematic Clusters

Concerning the first cluster, it pertains mainly to the psychological and cognitive aspects of cancer. Specifically, breast cancer survivors demonstrate objective deficits across a wide range of cognitive domains, including attention, executive functions, verbal and visuospatial skills, working memory, and motor skills [38]. Distressing symptoms such as anxiety, pain, feeling bloated, trouble sleeping, feelings of sadness and nervousness were present among newly diagnosed ovarian cancer (OC) patients, with anxiety being the most prominent one [30]. The authors also reported that cognitive impairment was present during early PC diagnosis, while patient symptomatology was related to both objective and subjective cognitive difficulties, possibly reflecting Asians’ disposition towards both neuropsychological disorders and assessment processes. This latter observation emphasizes the need to take into consideration patients’ ethnic background during evaluation processes. Reduced quality of life, depression, anxiety and cognitive disturbances among cancer patients have been reported by additional researchers, while depression was also associated with greater inflammation [22]. Psychological difficulties may also burden cognition. Depression among breast cancer survivors, for instance, predicted both poor objective and subjective cognitive performance, while depression coupled with increased inflammation and/or intestinal permeability led to an heightened likelihood of subjective cognitive difficulties, indicating greater awareness [24]. The observation of the above studies highlight, not only an intersection between psychological well-being and cognitive health, but also the need to perform careful and holistic assessments, and develop intervention programs tailored to each patient’s needs.

The second emerged cluster is circumscribed to the study of the neuropsychological effects of both cancer and its treatment strategies (e.g., chemotherapy) and its neurobiological indices, such as fMRI. Progressive neurofunctioning alterations have been observed among medulloblastoma underaged survivors. Medulloblastoma survivors have shown disruptions in brain areas responsible for visual processing and orthographic recognition (i.e., ventral visual stream and occipital regions) and difficulties in reading and phonological awareness, indicating the delayed consequences of cancer treatment on brain and cognitive functions [93]. Cancer- and/or chemotherapy-related cognitive deficits are a frequent aftermath among patients with Hodgkin lymphoma as well [94,95]. However, disease severity or chemotherapy intensity level did not seem to constitute risk factors of brain health and the level of cognitive impairment [96], mitigating physician concerns regarding treatment protocols. Pre-, and post-chemotherapy treatment comparisons among breast cancer survivors have indicated greater gray matter volume in various brain areas, namely frontal regions, insula and supramarginal gyrus after chemotherapy. Greater white matter volume (WMV) in the corpus callosum and the uncinate fasciculus (UF), and decreased WMV was also observed in the cerebellum and the occipital fusiform gyrus after chemotherapy in comparison to prior chemotherapy treatment. Moreover, the WMV alterations in the cerebellum and the UF over time were also related to increased subjective cognitive impairment [44]. The aforementioned findings suggest that, apart from the direct cancer consequences to cognitive functioning, cancer treatment, and more specifically chemotherapy, may also lead to neuropsychological deficits through its effects to various brain regions, raising the importance of long-term patient evaluation and cognitive rehabilitation.

Cluster three represents a convergence of surgical strategies, tumor classification frameworks, and neuropsychological evaluation methodologies aimed at optimizing treatment outcomes for slow-growing brain tumors. Central terms such as awake surgery, meningioma, diffuse low-grade glioma, and neuropsychological assessment situate this cluster firmly within an interdisciplinary landscape that bridges operative precision with the preservation of cognitive and functional quality of life. Within this thematic area, evidence underscores the dual challenge faced by clinicians: the imperative of maximal safe resection and the equally critical goal of maintaining neurocognitive integrity. For instance, Schouwenaars et al. [69] demonstrated that meningioma patients frequently exhibit persistent cognitive deficits pre- and post-operatively, suggesting that surgical intervention alone may not suffice to restore cognitive function. Barberis et al. [57] highlighted that subtle verbal fluency impairments post–awake surgery in diffuse low-grade glioma patients can meaningfully influence vocational reintegration, underlining the prognostic role of targeted neuropsychological measures. Complementary findings by Mann et al. [64] and Xie et al. [71] on low-grade epilepsy-associated neuroepithelial tumors reveal that early surgical intervention may prevent the progression of seizure-related cognitive decline, emphasizing the importance of timely surgical decision-making. Furthermore, Rimmer et al. [68] shed light on self-management barriers in lower-grade glioma patients, where cognitive deficits and seizure burden influence autonomy, treatment adherence, and psychosocial adjustment—elements crucial to long-term survivorship planning. From a neuroimaging and connectomic perspective, the preservation of structural and functional brain networks emerges as a determinant of cognitive outcome, with studies suggesting that disruption to key hubs such as the default mode and attention networks correlates with executive and memory deficits [97]. This aligns with broader evidence in the cluster pointing to the need for pre- and post-operative cognitive monitoring, not only for clinical decision-making but also for personalized rehabilitation strategies.

Cluster four reflects an intersection between intensive oncological therapies and their cognitive, neurological, and survival implications in aggressive brain and head-and-neck malignancies. The thematic emphasis on terms such as cognition, radiotherapy, neuro-oncology, glioblastoma, functional connectivity, and symptoms underscores a research focus that extends beyond tumor control to encompass treatment-induced neurological sequelae, functional brain alterations, and patient-reported quality-of-life outcomes. Within this cluster, high-grade gliomas serve as a dominant context for studying the delicate balance between aggressive oncological control and the preservation of cognitive integrity. For example, Ahmeti et al. [72] demonstrated that preoperative peritumoral edema in meningiomas is strongly associated with cognitive deficits and poorer pre- and postoperative functional status, while Ihrig et al. [76] documented that androgen deprivation therapy in metastatic prostate cancer—though systemic—can impair visuomotor processing speed and language, reinforcing the cross-disease relevance of cancer-related cognitive decline. Glioblastoma-specific work [98] illustrates the transient worsening of attention, memory, and perceptual processing in the early post-surgical phase, followed by partial recovery before adjuvant radiotherapy—highlighting a potential “window” for targeted cognitive rehabilitation. From a network neuroscience perspective, Friedrich et al. [97] linked post-treatment glioma patients’ cognitive performance to the structural integrity of distributed brain networks, particularly default mode and attention hubs, while Cali et al. [74] used functional connectivity mapping to show that hereditary tumor syndromes can induce widespread resting-state network disruptions correlating with neurocognitive deficits. This thematic scope also includes non-glioma contexts such as nasopharyngeal carcinoma, where radiotherapy’s neurotoxic effects on brainstem and cortical structures influence both neuropsychological functioning and patient-reported outcomes [90]. Similarly, Smith and Wang [85] point to the role of computer-assisted cognitive training in mitigating long-term deficits in cancer survivors, underscoring the translational potential of cognitive rehabilitation across aggressive tumor types.

### 4.3. Current Trends and Emerging Research Themes

The bibliometric mapping of the literature reveals a rapidly expanding research landscape on neuropsychological and cognitive outcomes in oncology, with three dominant and interlinked thematic areas. The first, and most prominent, research front centers on cancer-related cognitive impairment (CRCI), particularly in breast cancer survivors after chemotherapy. Studies consistently report deficits in attention, executive function, memory, and processing speed, often persisting months or years post-treatment [85,99]. Neuroimaging research has identified alterations in the dorsal attention network and frontoparietal systems that correlate with neuropsychological impairments [53]. These findings extend beyond breast cancer, as cognitive decline has been documented in colorectal cancer survivors [100] and in patients undergoing endocrine or hormonal therapy [40].

A second major theme addresses biological and systemic contributors to CRCI. This includes the impact of menopausal status on cognition in papillary thyroid carcinoma patients [92], the role of neuroinflammation and intestinal permeability in mediating depression-related cognitive deficits in breast cancer survivors [24], and the metabolic consequences of androgen deprivation therapy on hippocampal integrity in prostate cancer [40]. Such studies illustrate the growing integration of neuropsychology with endocrinology, neuroimmunology, and metabolic neuroscience to better define the mechanisms of CRCI.

The third domain involves rehabilitation and intervention strategies. Web-based cognitive training has shown promise in improving overall cognitive function and executive performance shortly after primary treatment [99], while music-enhanced cognitive training may yield additional quality-of-life benefits [85]. These interventions address an urgent need for scalable, accessible strategies to mitigate the functional impact of CRCI. In parallel, sleep and circadian rhythm disturbances have emerged as a distinct research priority. Protocols such as ICANSLEEP-1 are combining polysomnography, actigraphy, cortisol profiling, and neuroimaging to elucidate the interplay between sleep quality, circadian regulation, and cognition in breast cancer [36]. This reflects a broader shift toward multi-domain, longitudinal designs that can capture both behavioral and neurobiological trajectories.

Looking ahead, the emerging research themes are:

- Precision cognitive oncology, integrating biomarkers, genetic profiles, and neuroimaging signatures for individualized risk assessment and rehabilitation planning.
- Longitudinal, multimodal studies capturing the dynamic trajectory of CRCI across cancer types, treatments, and survivorship phases.
- Digital cognitive monitoring through computerized assessment and telehealth delivery to increase accessibility.
- Holistic survivorship care models embedding cognitive health alongside physical, emotional, and social wellbeing.

### 4.4. Methodological Considerations and Limitations

While this bibliometric analysis provides a valuable understanding, several methodological constraints must be recognized concerning the evolving intellectual structure of research on cancer-related cognitive and neuropsychological outcomes. A primary limitation involves researchers’ reliance upon one bibliographic database. Scopus offers quite thorough coverage even if it does not index all of the relevant journals. It also does not fully capture interdisciplinary research subtleties. Less frequently cited sources or region-specific publications may be underrepresented therefore especially those outside the Anglophone scientific community. The analysis also included solely English-disseminated refereed journal publications. Language bias could be taken into account, which curtails findings’ applicability throughout countries not English-speaking, considering emphasis upon inclusive, culturally sensitive research in cancer care. Author-defined keywords with publication metadata intrinsically impact the bibliometric clustering process. Precision as well as uniformity fluctuate within such descriptors and data. Certain studies might employ broad terminology such as “cognitive dysfunction,” whereas others denote particular conditions such as “chemobrain” or “executive deficits.” This variability can impact the way that researchers allocate studies toward thematic groupings and can diminish the granular domain-specific perceptions. Also, citation-based metrics, which are utilized to evaluate influence or centrality, do not explicitly denote research quality. These metrics do not directly reveal clinical impact either. Cited articles with frequency might focus on topic vogue or author eminence. The analysis does not assess the empirical validity, effect sizes, or reproducibility of findings across the literature, which remain essential components of evidence synthesis in clinical domains.

### 4.5. Directions for Future Research

Based upon the subject analysis, several key areas for future research emerge. First, additional conceptual perspicuity will be required as well as uniformity is additionally required. Even with increasing acknowledgement of CRCI, definitions, diagnostic criteria, and assessment methods are still variable. Upcoming research must seek to formulate agreement-derived structures which pinpoint, track, as well as group mental alterations amid varied cancer demographics alongside therapy phases. Longitudinal studies including iterative evaluations preceding, concurrent with, and succeeding intervention are crucially required. Most of the current publications are cross-sectional otherwise adhere to only a brief duration, which constrains how we perceive cognitive alteration’s path as well as how it progress over time. Also, efforts must be made for assimilation of cognitive side effects. Standard oncology clinical trials should be incorporated also. Cognitive decline possess substantial ramifications regarding functional autonomy, therapeutic compliance, and existence quality though they are often absent from principal outcomes or understated. Integrating verified cognitive endpoints will augment clinical research’s pertinence and utility.

Advanced neuroimaging with biomarker studies is of increasing importance. Transforming these mechanistic perceptions into concrete screening instruments and therapeutic objectives still exhibits difficulties. In order to unite this divergence, upcoming studies must address actions as well as clinically applicable indicators that are employable throughout practical contexts. Subsequently, increased consideration should be given toward susceptible and marginalized demographics, including senior patients, minority communities, as well as patients from underprivileged environments. For future studies, inclusive methodologies and stratified analyses should be employed to tackle these disparities since cognitive outcomes may diverge greatly among sociodemographic strata. Digital health technologies signify auspicious horizons ultimately. These technologies include wearable cognitive monitoring tools alongside ecological momentary assessments and cognitive rehabilitation platforms. Nonetheless, to ascertain their merit, extensibility, and moral quandaries, researchers should systematically examine them via experiments and analyses. One promising yet underrepresented direction is the role of the circadian clock in cancer and cognitive outcomes, which warrants dedicated bibliometric attention as the field evolves.

## 5. Conclusions

This bibliometric and science mapping analysis provides the first comprehensive quantitative overview of research on neuropsychological and cognitive outcomes in oncology over the past decade. The results reveal a steadily expanding and thematically diverse field, with four dominant research domains encompassing psychosocial and emotional dimensions, biological mechanisms, surgical and neuropsychological perspectives, and treatment-related cognitive effects in high-grade malignancies. The integration of neuroimaging, cognitive assessment, and patient-reported outcomes demonstrates an increasing methodological sophistication and a growing commitment to translational relevance. Despite significant progress, the field remains characterized by methodological heterogeneity and fragmented collaboration networks. These factors limit the generalizability of findings and call for more integrated and collaborative research approaches. Importantly, the identified trends—such as the focus on cancer-related cognitive impairment, mechanistic biomarker research, and scalable rehabilitation strategies—underscore a shift toward more personalized, patient-centered survivorship care. By mapping the intellectual structure and emerging research fronts, this review provides a strategic foundation for future investigations. Targeted efforts to unify methodological approaches, expand collaborative networks, and embed cognitive endpoints into oncology trials will be essential for advancing both scientific knowledge and clinical impact. In doing so, the field can move toward a cohesive agenda that not only elucidates the mechanisms of cancer-related cognitive change but also translates this knowledge into effective interventions, ultimately improving quality of life for cancer survivors worldwide.

## Figures and Tables

**Table 1:** Inclusion and exclusion criteria for article selection.

Inclusion Criteria	Exclusion Criteria
Peer-reviewed journal articles	Articles involving animal models (e.g., rats)
Written in English	Articles written in languages other than English
Published between 2015 and 2025	Case reports and Protocol papers
Focus on neuropsychological or cognitive deficits associated with cancer	Articles from non-academic sources (e.g., magazines, reports, conference abstracts)
Human adult populations	Studies involving children

**Table 2:** Overview of Science Mapping Techniques.

Technique	Unit of Analysis	Minimum Threshold	Weight (Node Size)	Visualization Type
Co-authorship	Organizations	2 documents	Number of documents	Network
Bibliographic Coupling	Sources	2 documents	Number of documents	Overlay
Co-citation	Authors	30 citations	Number of citations	Network
Co-occurrence	Author keywords	3 occurrences	Number of keyword occurrences	Network

**Table 3:** Top Authors Based on Number of Publications and Citations.

Author	Documents	Citations
Eustache, Francis	5	151
Giffard, Bénédicte	5	151
Joly, Florence	5	151
Rutten, Geert-Jan M.	4	122
Sitskoorn, Margriet M.	4	191
Araújo-Soares, Vera	4	23
Burns, Richéal	4	23
Chen, Vincent Chin-Hung	4	43
Dutton, Lizzie	4	23
Finch, Tracy	4	23
Gallagher, Pamela	4	23
Lewis, Joanne	4	23
Rimmer, Ben	4	23
Sharp, Linda	4	23
Williams, Sophie	4	23

**Table 4:** Top Sources Based on Number of Publications and Citations.

Source	Documents	Citations
Journal of Neuro-Oncology	9	194
Psycho-Oncology	6	99
Brain Imaging and Behavior	5	132
World Neurosurgery	5	86
BMJ Open	5	9
Cancers	5	32
BMC Cancer	4	32
Acta Neurochirurgica	4	89
Neuro-Oncology	4	234
Neuro-Oncology Practice	4	84
Frontiers in Oncology	4	50
Journal of Cancer Survivorship	4	20
Oncologist	3	141
Neuro-Oncology Advances	3	14
Brain Sciences	3	18
Supportive Care in Cancer	3	18

## Data Availability

No new data were created or analyzed in this study. Data sharing is not applicable to this article.

## Supplementary Materials

The following supporting information can be downloaded at https://www.mdpi.com/article/10.3390/medsci13030191/s1. Table S1. Included studies utilized for the analysis.
