# Supplementary material for: Mapping Cognitive Oncology: A Decade of Trends and Research Fronts
Source: Med Sci (Basel). 2025 Sep 15;13(3):191. doi: 10.3390/medsci13030191 (PMC12452770; doi:10.3390/medsci13030191)
Supplement: Supplementary file 1 [file medsci-13-00191-s001.zip › medsci-3845094-supplementary.pdf]

**Table S1.** Included studies utilized for the analysis.

| Authors | Title | Year | Author Keywords |
|---|---|---|---|
| Zheng Y.; Zhao J.; Shi Y.; Gui Z.; Xu C.; Wu Q.; Zhu L.; Wang Z.; Zhang H.; He L. | Impact of menopausal status on cognitive function in female papillary thyroid carcinoma patients: a longitudinal propensity score matched study | 2024 | Cognitive function; Longitudinal study; Menopausal women; Papillary thyroid cancer; Propensity score matching |
| Guo K.; Liu X.; Gong X.; Li A.; Liu Y.; Li X.; Zhou D.; Hong Z. | Autoimmune encephalitis with mGluR5 antibodies: A case series from China and review of the literature | 2023 | autoimmune encephalitis; China; metabotropic glutamate receptor 5; Ophelia syndrome; outcome |
| Dwek M.-R.; Newman S.P.; Brini S.; Holder P.; Machesney M.; Propper D.; Rixon L.R.; Hirani S.P.; Hurt C.S. | The impact of chemotherapy on cognitive performance post-surgery in patients with colorectal cancer: A prospective cohort study | 2023 | cancer; chemotherapy; cognition; colorectal; impairment; oncology; surgery |
| Shen C.-Y.; Tsai Y.-H.; Chen V.C.-H.; Chou M.-C.; McIntyre R.S.; Weng J.-C. | Comparison of functional dorsal attention network alterations in breast cancer survivors before and after chemotherapy | 2021 | Breast cancer; Chemotherapy; Dorsal attention network; Resting-state functional MRI |
| Padovan B.V.; Bijl M.A.J.; Langendijk J.A.; van der Laan H.P.; Van Dijk B.A.C.; Festen S.; Halmos G.B. | Evaluation of a new two-step frailty assessment of head and neck patients in a prospective cohort | 2024 | Adverse outcomes; Frailty; Geriatric screening; Head and neck oncology |
| Elia C.; de Girolamo L.; Clarisse B.; Galin M.; Rehel S.; Clochon P.; Doidy F.; Segobin S.; Viader F.; Naveau M.; Delcroix N.; Segura-Djezzar C.; Grellard J.-M.; Lequesne J.; Etard O.; Martin T.; Quarck G.; Eustache F.; Joly F.; Giffard B.; Perrier J. | Effects of sleep disturbances and circadian rhythms modifications on cognition in breast cancer women before and after adjuvant chemotherapy: the ICANSLEEP-1 protocol | 2023 | Adjuvant chemotherapy; Breast cancer; Circadian rhythms; Cognition; Sleep structure; White matter integrity |

| | | | |
|---|---|---|---|
| Gaynor A.M.; Ahsan A.; Jung D.; Schofield E.; Li Y.; Ryan E.; Ahles T.A.; Root J.C. | Novel computerized neurocognitive test battery is sensitive to cancer-related cognitive deficits in survivors | 2024 | Breast cancer; Cancer-related cognitive dysfunction; Cognitive impairment; Survivorship |
| Smith T.M.; Wang W. | Comparison of a standard computer-assisted cognitive training program to a music enhanced program: A mixed methods study | 2021 | behavioral science; breast cancer; complementary medicine; survival |
| Guo P.; Xing X.; Wu K.; Wang Y.; Chen Z.; Cao L.; Li X.; Li N. | 1H-MRS study of hippocampus in advanced prostate cancer patients: Relationship between hippocampal secondary damage and cognitive disorder following combined androgen blockade therapy | 2025 | |
| Lindberg M.F.; Deau E.; Arfwedson J.; George N.; George P.; Alfonso P.; Corrionero A.; Meijer L. | Comparative Efficacy and Selectivity of Pharmacological Inhibitors of DYRK and CLK Protein Kinases | 2023 | |
| Schouwenaars I.T.; de Dreu M.J.; Rutten G.-J.M.; Ramsey N.F.; Jansma J.M. | Cognitive brain activity before and after surgery in meningioma patients | 2024 | cognition; functional MRI; meningioma; tumour resection; working memory |
| Cali R.J.; Nephew B.C.; Moore C.M.; Chumachenko S.; Sala A.C.; Cintron B.; Luciano C.; King J.A.; Hooper S.R.; Giardiello F.M.; Cruz-Correa M. | Altered neural networks and cognition in a hereditary colon cancer | 2021 | Cancer; Cognition; Familial Adenomatous Polyposis; fMRI; Neural networks; Resting state functional connectivity |
| Mann C.; Conradi N.; Neuhaus E.; Konczalla J.; Freiman T.M.; Spyrantis A.; Weber K.; Harter P.; Rosenow F.; Strzelczyk A.; Schubert-Bast S. | Early Epilepsy Surgery in Benign Cerebral Tumors: Avoid Your ‘Low-Grade’ Becoming a ‘Long-Term’ Epilepsy-Associated Tumor | 2022 | cMRI; cognitive deficit; dysembryoplastic neuroepithelial tumor; ganglioglioma; neuropsychological outcome; seizure; seizure outcome |
| Barberis M.; Poisson I.; Prévost-Tarabon C.; Letrange S.; Froelich S.; Thirion B.; Mandonnet E. | Verbal fluency predicts work resumption after awake surgery in low-grade glioma patients | 2024 | Ability to return to work; Awake surgery; Diffuse low-grade glioma; Mild cognitive deficits; Verbal fluency task |

| | | | |
|---|---|---|---|
| Xie M.-G.; Qiao J.; Wang X.; Zhou J.; Guan Y.; Liu C.; Zhao M.; Li T.; Luan G. | The cognitive functions and seizure outcomes of patients with low-grade epilepsy-associated neuroepithelial tumors | 2022 | Brain tumor; Cognition; Epilepsy; Risk factor; Seizure outcome; Surgery |
| Madison A.A.; Andridge R.; Kantaras A.H.; Renna M.E.; Bennett J.M.; Alfano C.M.; Povoski S.P.; Agnese D.M.; Lustberg M.; Wesolowski R.; Carson W.E., III; Williams N.O.; Reinbolt R.E.; Sardesai S.D.; Noonan A.M.; Stover D.G.; Cherian M.A.; Malarkey W.B.; Kiecolt-Glaser J.K. | Depression, Inflammation, and Intestinal Permeability: Associations with Subjective and Objective Cognitive Functioning throughout Breast Cancer Survivorship | 2023 | attention; depression; executive function; inflammation; LBP; memory; mood |
| Kalita O.; Hrabalek L.; Halaj M.; Hok P.; Franc D.; Klementova Y.; Dolezel M.; Cechakova E.; Sporikova Z.; Drabek J.; Hajduch M.; Tuckova L. | Very late complications of oncotherapy in glioblastoma patients: A case series | 2022 | corticosteroid; glioblastoma; oncotherapy; stroke-like syndrome |
| Leonetti A.; Puglisi G.; Rossi M.; Viganò L.; Conti Nibali M.; Gay L.; Sciortino T.; Howells H.; Fornia L.; Riva M.; Cerri G.; Bello L. | Factors Influencing Mood Disorders and Health Related Quality of Life in Adults With Glioma: A Longitudinal Study | 2021 | adjuvant treatments; brain tumors; health related quality of life; mood disorders; recovery |
| van Kessel E.; Huenges Wajer I.M.C.; Ruis C.; Seute T.; Fonville S.; De Vos F.Y.F.L.; Verhoeff J.J.C.; Robe P.A.; van Zandvoort M.J.E.; Snijders T.J. | Cognitive impairments are independently associated with shorter survival in diffuse glioma patients | 2021 | Cognition; Diffuse glioma; Etiologic; Prognosis; Survival |

| | | | |
|---|---|---|---|
| Dalboni da Rocha J.L.; Zou Stinnett P.; Scoggins M.A.; McAfee S.S.; Conklin H.M.; Gajjar A.; Sitaram R. | Functional MRI Assessment of Brain Activity Patterns Associated with Reading in Medulloblastoma Survivors | 2024 | brain tumor; cognitive impairment; fMRI; medulloblastoma; rapid automatized naming; reading |
| Kleinknecht K.R.; Bierend M.; Keim L.-M.; Bartels F.; Lampit A.; Finke C. | Computerized cognitive training improves cognitive function in primary breast cancer survivors | 2024 | |
| Ahmeti H.; Caliebe A.; Röcken C.; Jansen O.; Mehdorn M.H.; Synowitz M. | Impact of peritumoral brain edema on pre- and postoperative clinical conditions and on long-term outcomes in patients with intracranial meningiomas | 2023 | Brain edema; Intracranial meningiomas; Long-term outcomes; Meningioma surgery; Neurological conditions |
| Jung W.H.; Chun M.; Kim N.H. | Structural brain changes and associated self-perceived cognitive deficits in chemotherapy-exposed patients with breast cancer: a longitudinal T1 and DTI study | 2024 | Brain structure; Breast cancer; Chemotherapy; Gray matter; Subjective cognitive function; White matter |
| Saadullah M.; Farid A.; Ali A.; Rashad M.; Naseem F.; Rashid S.A.; Ghazanfar S.; Yasin M.; Akhtar N.; Almuhayawi M.S.; Alruhaili M.H.; Selim S. | Molecular Modeling Study of Novel Lancifolamide Bioactive Molecule as an Inhibitor of Acetylcholinesterase (AChE), Herpes Simplex Virus (HSV-1), and Anti-proliferative Proteins | 2022 | anti-Alzheimer's; anti-proliferative; anti-viral; antibiotic-resistant bacteria; combretaceae; Conocarpus lancifolius; cytotoxic; infection; lancifolamide |
| Couderc A.-L.; Tomasini P.; Greillier L.; Nouguerède E.; Rey D.; Montegut C.; Thomas P.-A.; Barlesi F.; Villani P. | Functional status in older patients with lung cancer: an observational cohort study | 2022 | Aged; Cancer treatment protocols; Daily living activities; Functional status; Lung cancer; Mortality |
| Datta A.; Roy S. | Social Emotion Recognition, Social Functioning and Suicidal Behaviour in Breast Cancer Patients in India | 2022 | |
| Rimmer B.; Balla M.; Dutton L.; Williams S.; Araújo-Soares V.; Gallagher P.; Finch T.; Lewis J.; Burns R.; Menger F.; Sharp L. | Barriers and facilitators to self-management in people living with a lower-grade glioma | 2024 | Barriers; Facilitators; Lower-grade glioma; Self-management |

| | | | |
|---|---|---|---|
| Spoor J.K.H.; Donders-Kamphuis M.; Veenstra W.S.; van Dijk S.A.; Dirven C.M.F.; Sillevis Smitt P.A.E.; van den Bent M.J.; Leenstra S.; Satoer D.D. | Cognition and health-related quality of life in long-term survivors of high-grade glioma: an interactive perspective from patient and caregiver | 2024 | Caregivers; Cognition; High-grade glioma; Long-term survival; Quality of life |
| Gomaa A.A.; Abdallah D.M.; El-Abhar H.S.; El-Mokadem B.M. | Repurposing Aprepitant: Can it protect against doxorubicin-induced Chemobrain beyond its antiemetic role? | 2024 | Aprepitant; Cognitive dysfunction; ER stress; miR-146a; PERK/eIF-2α/ATF-4/CHOP; Substance P |
| Friedrich M.; Filss C.P.; Lohmann P.; Mottaghy F.M.; Stoffels G.; Weiss Lucas C.; Ruge M.I.; Shah N.J.; Caspers S.; Langen K.-J.; Fink G.R.; Galldiks N.; Kocher M. | Structural connectome-based predictive modeling of cognitive deficits in treated glioma patients | 2024 | brain networks \| cognitive functions \| diffusion-weighted imaging \| glioma \| structural connectivity |
| Durán-Gómez N.; López-Jurado C.F.; Nadal-Delgado M.; Pérez-Civantos D.; Guerrero-Martín J.; Cáceres M.C. | Chemotherapy-Related Cognitive Impairment in Patients with Breast Cancer Based on Functional Assessment and NIRS Analysis | 2022 | breast neoplasm; cerebral blood flow; cognitive impairment; dorsolateral prefrontal cortex; near-infrared spectroscopy |
| Wang L.; Ding Y.; Zhang Y.; Chen Y.; Xue M.; Wang X. | The association between neuropsychological impairment, self-perceived cognitive deficit, symptoms, and health related quality of life in newly diagnosed ovarian cancer patients | 2024 | Health related quality of life; Neuropsychological impairment; Newly diagnosed ovarian cancer; Self-perceived cognitive deficit; Symptoms |
| Sinha R.; Masina R.; Morales C.; Burton K.; Wan Y.; Joannides A.; Mair R.J.; Morris R.C.; Santarius T.; Manly T.; Price S.J. | A Prospective Study of Longitudinal Risks of Cognitive Deficit for People Undergoing Glioblastoma Surgery Using a Tablet Computer Cognition Testing Battery: Towards Personalized Understanding of Risks to Cognitive Function | 2023 | cognition; deficits; glioblastoma; neuro-oncology; quality of life; risks; surgery; survivorship |
| Ihrig A.; Pernt P.M.; Zschäbitz S.; Huber J.; Friederich H.-C.; Bugaj T.J.; Maatouk I. | Neurocognitive effects of androgen deprivation therapy and new hormonal agents in a sample of patients with metastatic prostate cancer | 2023 | Androgen deprivation therapy; Hormonal agents; Metastatic prostate cancer; Neurocognitive effects |

| | | | |
|---|---|---|---|
| Lin R.J.; Owens C.N.; Drill E.; Iannotta A.; Oliveros M.; Schick D.L.; Noy A.; Gerecitano J.F.; Drullinsky P.R.; Caron P.C.; Kumar A.; Matasar M.J.; Moskowitz C.; Korc-Grodzicki B.; Zelenetz A.D.; Salles G.A.; Hamlin P.A. | Prephase rituximab/prednisone therapy and aging-related, proinflammatory cytokine milieu in older, vulnerable patients with newly diagnosed diffuse large B-cell lymphoma | 2022 | |
| Stapleton S.; Darlington A.-S.E.; De Bono J.S.; Wiseman T. | What is the impact of targeted therapies given within phase i trials on the cognitive function of patients with advanced cancer: A mixed-methods exploratory study conducted in an early clinical trials unit | 2022 | adult oncology; clinical pharmacology; clinical trials; delirium & cognitive disorders |
| Vooijs M.; Robertson F.C.; Blitz S.E.; Jungk C.; Krieg S.M.; Schucht P.; Vleeschouwer S.D.; Vincent A.J.P.E.; Berger M.S.; Nahed B.V.; Broekman M.L.D.; Gerritsen J.K.W. | Level I and II deficits—A clinical survey on international practice of awake craniotomy and definitions of postoperative “major” and “minor” deficits | 2024 | awake craniotomy; complications; glioma; neurological morbidity; survey |
| Buunk A.M.; Gerritsen M.J.J.; Jeltema H.-R.; Wagemakers M.; Metzemaekers J.D.M.; Groen R.J.M.; Spikman J.M. | Emotion Recognition in Patients with Low-Grade Glioma before and after Surgery | 2022 | brain tumor; emotion recognition; low-grade glioma; social cognition |
| West T.; Cavallero C.; Ceccherini R.; Foladore S.; Generali D.; Versace F.; Scaggiante B. | Impact of psychosocial, behavioral and lifestyle factors on subjective cognitive complaints and perceived quality of life in a large cohort of Italian breast cancer patients | 2022 | adjuvant therapies; breast cancer; cancer-related cognitive impairment; chemotherapy; cognitive reserve; sleep quality; subjective cognitive complaints |
| Jiang C.; Zhu M.; Wei D.; Duan H.; Zhang Y.; Feng X. | SCLC and anti-GABABR encephalitis: A retrospective analysis of 60 cases in China | 2022 | anti-GABABR encephalitis; limbic encephalitis; small cell lung cancer |
| Luckett P.H.; Olufawo M.O.; Park K.Y.; Lamichhane B.; Dierker D.; Verastegui G.T.; Lee | Predicting post-surgical functional status in high-grade glioma with resting state fMRI and machine learning | 2024 | Brain tumor; Functional MRI; High-grade glioma; Machine learning |

| | | | |
|---|---|---|---|
| J.J.; Yang P.; Kim A.; Butt O.H.; Chheda M.G.; Snyder A.Z.; Shimony J.S.; Leuthardt E.C. | | | |
| Jung M.S.; Visovatti M.; Kim M.; Cha K.; Dlamini N.; Cui X. | Cognitive impairment in women newly diagnosed with thyroid cancer before treatment | 2022 | Cognitive changes; Symptoms; Thyroid cancer |
| Ng S.; Lemaitre A.-L.; Moritz-Gasser S.; Herbet G.; Duffau H. | Recurrent Low-Grade Gliomas: Does Reoperation Affect Neurocognitive Functioning? | 2022 | Awake surgery; Brain mapping; Low-grade glioma; Neuropsychological assessment; Quality of life; Reoperation; Tumor recurrence |
| Popp I.; Rau A.; Kellner E.; Reisert M.; Fennell J.T.; Rothe T.; Nieder C.; Urbach H.; Egger K.; Grosu A.L.; Kaller C.P. | Hippocampus-Avoidance Whole-Brain Radiation Therapy Is Efficient in the Long-Term Preservation of Hippocampal Volume | 2021 | atrophy; cognitive function; hippocampus; MRI; WBRT (whole-brain radiation therapy) |
| Lala M.-F.; Stadelmaier N.; Paternostre B. | Identification of the informal caregivers' suffering during cancer advanced palliative phase at home; [Repérage de la souffrance des aidants lors de la phase palliative avancée de cancer à domicile] | 2022 | Aidant proche; Family caregiver; Home nursing; Palliative care; Soins palliatifs; Soins à domicile; Souffrance; Suffering |
| Mundell N.; Owen P.J.; Dalla Via J.; Macpherson H.; Daly R.; Fraser S. | Does androgen deprivation impact associations between cognition and strength, fitness and function in community-dwelling men with prostate cancer? A cross-sectional study | 2021 | clinical physiology; delirium & cognitive disorders; neurological oncology; rehabilitation medicine; sports medicine; urological tumours |
| Schoenberg P.; Wulff-Burchfield E.; Schlundt D.; Bonnet K.; Dietrich M.; Murphy B. | Qualitative Classification of Late Systemic Symptoms in Head and Neck Cancer Survivors | 2024 | cancer survivorship; emotional disturbance; fatigue; head and neck cancer; pain; social support; spirituality; systemic symptoms |
| Zhu H.; Lin R.; Wang J.; Ruan S.; Hu T.; Lei Y.; Ke X.; Luo H. | Cognitive function and its associated factors among patients with cancer pain: A multicentre cross-sectional study in China | 2024 | Cancer pain; Delirium & cognitive disorders; Pain management; PAIN MANAGEMENT |
| Kocher M.; Jockwitz C.; Lohmann P.; Stoffels G.; Filss C.; Mottaghy F.M.; Ruge M.I.; Lucas C.W.; Goldbrunner R.; | Lesion-function analysis from multimodal imaging and normative brain atlases for prediction of cognitive deficits in glioma patients | 2021 | Brain networks; Cognitive testing; Glioma; Positron emission tomography; Radiotherapy |

| | | | |
|---|---|---|---|
| Shah N.J.; Fink G.R.; Galldiks N.; Langen K.-J.; Caspers S. | | | |
| Sideroff S.; Wellisch D.; Yarema V. | A neurotherapy protocol to remediate cognitive deficits after adjuvant chemotherapy: A pilot study | 2023 | breast cancer; chemobrain; cognitive deficits; EEG; EEG biofeedback; neurofeedback |
| Oort Q.; Dirven L.; Sikkes S.A.M.; Aaronson N.; Boele F.; Brannan C.; Egeter J.; Grant R.; Klein M.; Lips I.; Narita Y.; Sato H.; Sztankay M.; Stockhammer G.; Talacchi A.; Uitdehaag B.M.J.; Reijneveld J.C.; Taphoorn M.J.B. | Development of an EORTC questionnaire measuring instrumental activities of daily living (IADL) in patients with brain tumours: phase I–III | 2021 | Brain tumour; Daily functioning; IADL; Instrumental activities of daily living; Questionnaire |
| Fortes G.N.; Fortes M.F.; Fortes M.N.; Gomes F.C.; Seelaendar M.C.L.; de Pinho A.M.S.; de Jesus J.D.C.R.; Otoch J.P. | Mood, Anxiety, and Cognitive Alterations in Cancer Patients | 2024 | cachexia; humor disorders; inflammation; neoplasia |
| de Sain A.M.; Mantione M.H.M.; Wajer I.M.C.H.; van Zandvoort M.J.E.; Willems P.W.A.; Robe P.A.; Ruis C. | A timeline of cognitive functioning in glioma patients who undergo awake brain tumor surgery | 2023 | Brain tumor; Cognition; Craniotomy; Glioma |
| Rimmer B.; Balla M.; Dutton L.; Lewis J.; Brown M.C.; Burns R.; Gallagher P.; Williams S.; Araújo-Soares V.; Finch T.; Menger F.; Sharp L. | Identifying and understanding how people living with a lower-grade glioma engage in self-management | 2024 | Lower-grade glioma; Qualitative; Self-management; Wellbeing |
| Abraham E.H.; Khan B.; Ling E.; Bernstein L.J. | The Development and Evaluation of a Patient Educational Resource for Cancer-Related Cognitive Dysfunction | 2022 | Cancer survivorship; Cancer-related brain fog; Cancer-related cognitive impairment; Chemobrain; Patient education; Symptom management |

| | | | |
|---|---|---|---|
| Bartels F.; Wandrey M.-M.; Aigner A.; Strönisch T.; Farmer K.; Rentzsch K.; Tessmer A.; Grohé C.; Finke C. | Association between Neuronal Autoantibodies and Cognitive Impairment in Patients with Lung Cancer | 2021 | |
| Baghdadli A.; Arcuri G.G.; Green C.G.; Gauthier L.R.; Gagnon P.; Gagnon B. | The Fast Cognitive Evaluation (FaCE): a screening tool to detect cognitive impairment in patients with cancer | 2023 | Brain fog; Cancer; Chemo brain; Cognition; Memory; Mild cognitive impairment |
| Muthumuni D.; Scott I.; Chochinov H.M.; Mahar A.L.; Garland S.N.; Schulte F.; Lambert P.; Lix L.; Garland A.; Oberoi S. | Feasibility and Acceptability of a Virtual ‘‘Coping with Brain Fog’’ Intervention for Improving Cognitive Functioning in Young Adults with Cancer | 2023 | cancer survivors; cognition; intervention; mental fatigue; neoplasms |
| Motah M.; Gams Massi D.; Fouda Bekolo F.; Akweseh Nju N.; Ndoumbe A.; Moumi M.; Sango A.; Shu P.; Eyenga V. | Epidemiological profile of brain tumors in Cameroon: a retrospective study | 2021 | Astrocytoma; Brain tumors; Cameroon; Epidemiology; Meningioma; Pituitary adenoma |
| Bruhn H.; Tavelin B.; Rosenlund L.; Henriksson R. | Do presenting symptoms predict treatment decisions and survival in glioblastoma? Real-world data from 1458 patients in the Swedish brain tumor registry | 2024 | cognition; glioblastoma; prognostic factors; survival; symptoms |
| Boelsbjerg H.B.; Kurita G.P.; Sjøgren P.; Hansen N.V. | Combining subjective and objective appraisals of cognitive dysfunction in patients with cancer: a deeper understanding of meaning and impact on suffering? | 2022 | Cognitive dysfunction; Existential well-being; Neoplasms; Neuropsychological tests; Patient-reported outcome measures; Qualitative research |
| Laaker C.J.; Cantelon C.; Davis A.B.; Lloyd K.R.; Agyeman N.; Hiltz A.R.; Smith B.L.; Konsman J.P.; Reyes T.M. | Early life cancer and chemotherapy lead to cognitive deficits related to alterations in microglial-associated gene expression in prefrontal cortex | 2023 | |
| Megari K. | Neuropsychological functioning among patients with different types of cancer: Postchemotherapy cognitive impairment and implications for rehabilitation; [Neuropsychologische Funktionen bei Patienten mit | 2021 | Cancer; Cognitive domains; Cognitive enhancement; Neuropsychological assessment; |

| | | | |
|---|---|---|---|
| | verschiedenen malignen Tumoren: Kognitive Beeinträchtigung nach Chemotherapie und Implikationen für die Rehabilitation] | | Neuropsychological functioning; Postchemotherapy cognitive impairment |
| González G.M.C.; Rosero E.V.; Franco-Rocha O.Y.; Piraneque J.L.F.; Castro A.M.; Castellanos M. | Cultural adaptation and validity of the memorial symptom assessment scale in adults with cancer in Colombia | 2022 | Colombia; neoplasms; psychometrics; symptom assessment; validation study |
| Crooms R.C.; Nnemnbeng J.F.; Taylor J.W.; Goldstein N.E.; Vickrey B.G.; Gorbenko K. | "Life…Gets Turned Upside-Down…" Opportunities to Improve Palliative Care for High-Grade Glioma | 2024 | Glioma; Neuro-oncology; Neuropalliative care; Palliative care delivery models; Qualitative theme analysis |
| Vayyat S.; Revand R.; Rastogi S.; Sharma R.; Kaur S. | Patient-reported cognitive impairments and objective neuropsychological deficits in young sarcoma patients undergoing chemotherapy and its comparison to healthy controls: A tertiary health care study from India | 2024 | cancer rehabilitation; chemotherapy; cognitive impairments; sarcoma; young adults |
| Fayette D.; Juríčková V.; Kozák T.; Mociková H.; Gaherová L.; Fajnerová I.; Horáček J. | Cognitive impairment associated with Hodgkin's lymphoma and chemotherapy | 2023 | Cancer; Chemotherapy; Chemotherapy-related cognitive deficit; Hodgkin's lymphoma; Neuropsychology |
| Bai X.; Zheng J.; Zhang B.; Luo Y. | Cognitive Dysfunction and Neurophysiologic Mechanism of Breast Cancer Patients Undergoing Chemotherapy Based on Resting State Functional Magnetic Resonance Imaging | 2021 | Breast cancer; Chemotherapy; Cognitive impairment; Diffusion tensor imaging; Resting state functional magnetic resonance |
| Zilioli A.; Misirocchi F.; Mutti C.; Pancaldi B.; Mannini E.; Spallazzi M.; Parrino L.; Cerasti D.; Michiara M.; Florindo I. | Volumetric hippocampal changes in glioblastoma: a biomarker for neuroplasticity? | 2023 | Glioblastoma; Hippocampus; Neuroplasticity; VBM |
| Wang J.; Zhang H.; Augenreich M.; Martinez-Lemus A L.; Liu Z.; Kang X.; Lu B.; Chang H.-M.; Yeh E.T.H.; Cata J.; Rangaraju S.; Wulff H.; Li D.-P. | Microglia-Mediated Synaptic Dysfunction Contributes to Chemotherapy-Related Cognitive Impairment | 2025 | chemotherapy; cognitive deficits; long-term potentiation; microglia; synaptic transmission |

| | | | |
|---|---|---|---|
| Kivioja T.; Posti J.P.; Sipilä J.; Rauhala M.; Frantzén J.; Gardberg M.; Rahi M.; Rautajoki K.; Nykter M.; Vuorinen V.; Nordfors K.; Haapasalo H.; Haapasalo J. | Motor dysfunction as a primary symptom predicts poor outcome: multicenter study of glioma symptoms | 2023 | brain neoplasm; cognitive disorder; epilepsy; glioma; headache; motor dysfunction; prognosis; symptoms |
| Fayette D.; Juríčková V.; Fajnerová I.; Horáček J.; Kozák T. | The Effect of Chemotherapy Treatment on Cognitive Impairment and Clinical Symptoms in Hodgkin Lymphoma Patients | 2025 | cancer; cancer-related cognitive impairment; chemotherapy; chemotherapy-related cognitive impairment; cognitive functions; Hodgkin lymphoma |
| Lemaitre A.-L.; Herbet G.; Ng S.; Moritz-Gasser S.; Duffau H. | Cognitive preservation following awake mapping-based neurosurgery for low-grade gliomas: A longitudinal, within-patient design study | 2022 | awake surgery; cognitive assessment; diffuse low-grade glioma; longitudinal assessment |
| Derks J.; Kulik S.D.; Numan T.; De Witt Hamer P.C.; Noske D.P.; Klein M.; Geurts J.J.G.; Reijneveld J.C.; Stam C.J.; Schoonheim M.M.; Hillebrand A.; Douw L. | Understanding Global Brain Network Alterations in Glioma Patients | 2021 | functional connectivity; glioma; magnetoencephalography; neuro-oncology; resting-state |
| Juríčková V.; Fayette D.; Jonáš J.; Fajnerová I.; Kozák T.; Horáček J. | Pretreatment Cancer-Related Cognitive Impairment in Hodgkin Lymphoma Patients | 2023 | affective distress; cancer-related cognitive impairment; Hodgkin lymphoma; neuropsychology; quality of life |
| Rimmer B.; Balla M.; Dutton L.; Williams S.; Lewis J.; Gallagher P.; Finch T.; Burns R.; Araújo-Soares V.; Menger F.; Sharp L. | "It changes everything": Understanding how people experience the impact of living with a lower-grade glioma | 2024 | lower-grade glioma; qualitative; quality of life; supportive care needs |
| Hussein S.A.; Tolba M.F.; Michel H.E.; Albohy A.; Azab S.S. | In silico and In vivo protective effect of biochanin-A mitigating doxorubicin-induced cognitive deficits and neuroinflammation: Insights to the role of p-Tau and miR-132 | 2025 | BIO-A; Chemobrain; DOX; MiR-132; SIRT1; Tauopathy |

| | | | |
|---|---|---|---|
| Ma Y.; Liu N.; Wang Y.; Zhang A.; Zhu Z.; Zhang Z.; Li Y.; Jian G.; Fu G.; Dong M.; Zheng G.; Zhu P.; Zhong G.; Bai S.; Chen S.; Wei X.; Tan J.; Wang X. | Cognitive adverse events in patients with lung cancer treated with checkpoint inhibitor monotherapy: a propensity score-matched analysis | 2023 | Checkpoint inhibitor; Cognitive adverse events; Immune-related adverse events; Minimal clinically important difference; Propensity score matching |
| Veronese S.; Bertocchi E.; Lissoni B.; Rudà R.; Silvani A.; Simonetti G.; Pisanello A.; Ieraci S.; Salmaggi A.; Merli R.; Verza M.; De Panfilis L.; Solari A.; Pace A. | Patient and carer involvement in the formulation of research questions: findings from the Italian guideline on palliative care in adults with glioma | 2023 | Brain tumors; Consumer involvement; Gliomas; Palliative care; Practice guideline; Qualitative study |
| Caponnetto P.; Schilirò G.; Maglia M.; Prezzavento G.C.; Baeken C.; Quattropani M.C. | Psychological and neuropsychological clinical impact in brain cancer patients and evidence-based psychological intervention: a systematic review of the literature | 2024 | Brain cancer; Cognitive-behavioural treatments; eHealth; Neuropsychology; Psychological disorders |
| Li H.; Wang C.; Guo C. | Post-marketing safety of lorlatinib: a real-world study based on the FDA adverse event reporting system | 2024 | adverse event; disproportionality; FAERS; lorlatinib; pharmacovigilance |
| van Grinsven E.E.; Cialdella F.; Verhoeff J.J.C.; Philippens M.E.P.; van Zandvoort M.J.E. | Different profiles of neurocognitive functioning in patients with brain metastases prior to brain radiotherapy | 2023 | brain metastases (BrMs); brain tumor; cancer; cluster analysis; cognition; neurocognitive functioning; neuropsychology; oncology; radiotherapy |
| Sanchez L.; Fernandez N.; Calle A.P.; Ladera V.; Casado I.; Bayon E.; Garcia I.; Sahagun A.M. | Assessing the Expression of Emotions in Breast Cancer Survivors during the Time of Recovery: Perspective from Focus Groups | 2022 | breast cancer; focus groups; information needs; long-term care; survivors; women’s health |
| Giovagnoli A.R.; Meneses R.F.; Paterlini C.; Silvani A.; Boiardi A. | Cognitive awareness after treatment for high-grade glioma | 2021 | Affective status; Cognitive awareness; High-grade glioma; Neuropsychological performance; Perceived cognitive abilities |

| | | | |
|---|---|---|---|
| Mahmoud A.M.A.; Mantawy E.M.; Wahdan S.A.; Ammar R.M.; El-Demerdash E. | Vildagliptin restores cognitive function and mitigates hippocampal neuronal apoptosis in cisplatin-induced chemo-brain: Imperative roles of AMPK/Akt/CREB/ BDNF signaling cascades | 2023 | AMPK/Akt/CREB; Chemo brain; Cisplatin; Cognitive function; Vildagliptin |
| Walker H.; Rimmer B.; Dutton L.; Finch T.; Gallagher P.; Lewis J.; Burns R.; Araújo-Soares V.; Williams S.; Sharp L. | Experiences of work for people living with a grade 2/3 oligodendroglioma: A qualitative analysis within the Ways Ahead study | 2023 | neurological oncology; occupational & industrial medicine; qualitative research |
| Guo Y.; Liu S.; Yan F.; Yin N.; Ni J.; Li C.; Pan X.; Ma R.; Wu J.; Li S.; Li X. | Associations between disrupted functional brain network topology and cognitive impairment in patients with rectal cancer during chemotherapy | 2022 | chemotherapy; cognitive impairment; graph theory analysis; rectal cancer; resting-state functional magnetic resonance imaging |
| Hajj A.; Khoury R.; Hachem R.; Awad A.; Hallit S.; Sacre H.; Nasr F.; Karak F.E.; Chahine G.; Kattan J.; Khabbaz L.R. | Clinical and genetic factors associated with self-reported cognitive deficits in women with breast cancer: the “CAGE-Cog” study | 2022 | Breast cancer; Chemotherapy; Cognitive function; FACT-Cog; OPRM1; Pharmacogenetics |
| Wang D.; Wang T.; Zhu M.; Sun J.; Zhou Z.; Chen J.; Teng L. | A Preliminary Study on the Relationship between Serum Heparan Sulfate and Cancer-Related Cognitive Impairment: The Moderating Role of Oxidative Stress in Patients with Colorectal Cancer | 2022 | cancer-related cognitive impairment; colorectal cancer; GSH; heparan sulfate; oxidative stress |
| Van Kessel E.; Krijnen E.A.; Ijpelaar S.; Huenges Wajer I.M.C.; Ruis C.; Seute T.; De Vos F.Y.F.L.; Verhoeff J.J.C.; Robe P.A.; Van Zandvoort M.J.E.; Snijders T.J. | Complications, compliance, and undertreatment do not explain the relationship between cognition and survival in diffuse glioma patients | 2022 | Cognition; Diffuse glioma; Mediation-Analyses; Survival |
| Raj J.A.T.; Shah J.; Ghanekar S.; John G.; Goda J.S.; Chatterjee A. | Pharmacological and therapeutic innovation to mitigate radiation-induced cognitive decline (RICD) in brain tumor patients | 2025 | Neuroinflammation; Pharmacotherapeutics; Phytochemicals; Radiation-induced cognitive decline |
| Seto T.; Nakane S.; Ji L.; Ueda Y.; Sugano H.; Takei N.; | Real-world safety and effectiveness of entrectinib in Japanese patients with ROS1 gene fusion-positive, unresectable, advanced/recurrent non-small cell lung cancer: Post-marketing surveillance | 2025 | Entrectinib; Japan; Non-small-cell lung cancer; Post-marketing surveillance; ROS1 fusion gene; ROS1 tyrosine kinase inhibitors |

| | | | |
|---|---|---|---|
| Kobayashi M.; Murayama A.; Yamamoto N. | | | |
| Dahouri A.; Sahebihagh M.H.; Gilani N. | Predictive factors associated with health-related quality of life in patients with colorectal cancer in Iran: A cross-sectional study | 2024 | Aging; Chronic Disease; Gastrointestinal tumours; ONCOLOGY; Public health; Risk Factors |
| Alexander J.F.; Mahalingam R.; Seua A.V.; Wu S.; Arroyo L.D.; Hörbelt T.; Schedlowski M.; Blanco E.; Kavelaars A.; Heijnen C.J. | Targeting the Meningeal Compartment to Resolve Chemobrain and Neuropathy via Nasal Delivery of Functionalized Mitochondria | 2022 | chemotherapy; cognition; encapsulation; intranasal; meninges; pain; stem cells |
| Pedras R.N.; Manhães M.F.M.; Carneiro A.M.; Okuma G.Y.; Elias S.; De Domenico E.B.L.; Bergerot C.D. | Assessment of Cognitive Impairment in Survivors of Breast Cancer: A Cross-sectional Study; [Avaliação de Prejuízo Cognitivo em Sobreviventes de Câncer de Mama: Estudo Transversal] | 2022 | Breast cancer; Cognitive dysfunction; Psycho-oncology; Survivors |
| Wang B.; Bie Z.; Wang X.; Tang H.; Liu P. | Characteristics of Perioperative Cognitive and Affective Function in Patients with Somatotroph Adenoma | 2023 | Affective; Cognitive; Growth hormone; Somatotroph adenomas; Surgery |
| Parsons M.W.; Peters K.B.; Floyd S.R.; Brown P.; Wefel J.S. | Preservation of neurocognitive function in the treatment of brain metastases | 2021 | brain neoplasms; cancer; cognition; neuropsychology; survivors |
| Brown K.; Sedig L.K.; Oswald-Mccloskey K.; Wang Y.; Stuchell E.; Jasty-Rao R. | Neuropsychologic Resource Utilization in Solid Tumor Survivors at a Single Institution: A Retrospective Chart Review | 2025 | education; neuropsychology; oncology; survivorship |
| Voon N.S.; Manan H.A.; Yahya N. | Remote assessment of cognition and quality of life following radiotherapy for nasopharyngeal carcinoma: deep-learning-based predictive models and MRI correlates | 2024 | Cognition; Deep neural network; Nasopharyngeal carcinoma; Radiotherapy; Remote |
| Sleight A.G.; Myers J.S.; Cook-Wiens G.; Baynes R.; Jo M.-Y.; Asher A. | Loneliness as a risk factor for cancer-related cognitive impairment: a secondary data analysis from the Haze study | 2023 | cancer; cancer rehabilitation; cognitive impairment; cognitive rehabilitation; Loneliness; oncology |

| | | | |
|---|---|---|---|
| Kam J.W.Y.; Brenner C.A.; Handy T.C.; Boyd L.A.; Liu-Ambrose T.; Lim H.J.; Hayden S.; Campbell K.L. | Sustained attention abnormalities in breast cancer survivors with cognitive deficits post chemotherapy: An electrophysiological study | 2016 | Adjuvant chemotherapy; Breast cancer; Cognitive deficits; Mind wandering; P300 ERP; Rest EEG; Sustained attention |
| Gullett J.M.; Cohen R.A.; Yang G.S.; Menzies V.S.; Fieo R.A.; Kelly D.L.; Starkweather A.R.; Jackson-Cook C.K.; Lyon D.E. | Relationship of fatigue with cognitive performance in women with early-stage breast cancer over 2 years | 2019 | breast cancer; cancer; chemobrain; chemotherapy; cognition; fatigue; longitudinal; oncology |
| Mo C.; Lin H.; Fu F.; Lin L.; Zhang J.; Huang M.; Wang C.; Xue Y.; Duan Q.; Lin W.; Chen X. | Chemotherapy-induced changes of cerebral activity in restingstate functional magnetic resonance imaging and cerebral white matter in diffusion tensor imaging | 2017 | Breast cancer; Chemotherapy; Cognitive disorder; Diffusion tensor imaging; Resting-state functional magnetic resonance imaging |
| Henderson F.M.E.; Cross A.J.; Baraniak A.R. | ‘A new normal with chemobrain’: Experiences of the impact of chemotherapy-related cognitive deficits in long-term breast cancer survivors | 2019 | breast cancer; cancer; chemobrain; cognitive impairment; illness perception; interpretative phenomenological analysis; qualitative methods; quality of life |
| Jung M.S.; Zhang M.; Askren M.K.; Berman M.G.; Peltier S.; Hayes D.F.; Therrien B.; Reuter-Lorenz P.A.; Cimprich B. | Cognitive dysfunction and symptom burden in women treated for breast cancer: a prospective behavioral and fMRI analysis | 2017 | Attention; Cognitive disorders; Functional magnetic resonance imaging; Short-term memory; Symptom assessment |
| Lange M.; Heutte N.; Rigal O.; Noal S.; Kurtz J.-E.; Lévy C.; Allouache D.; Rieux C.; Lefel J.; Clarisse B.; Veyret C.; Barthélémy P.; Longato N.; Castel H.; Eustache F.; Giffard B.; Joly F. | Decline in cognitive function in older adult swith early-stage breast cancer after adjuvant treatment | 2016 | |
| Li W.; Zhao J.; Ding K.; Chao H.H.; Li C.-S.R.; Cheng H.; Shen L. | Catechol-O-Methyltransferase gene polymorphisms and the risk of chemotherapy-induced prospective memory impairment in breast cancer patients with varying tumor hormonal receptor expression | 2020 | Adjuvant; Breast Neoplasms; Chemotherapy; Cognitive Therapy; Genetic; Male; Polymorphism |

| | | | |
|---|---|---|---|
| Chen V.C.-H.; Lin T.-Y.; Yeh D.-C.; Chai J.-W.; Weng J.-C. | Functional and structural connectome features for machine learning chemo-brain prediction in women treated for breast cancer with chemotherapy | 2020 | Breast cancer; Chemo brain; Connectome; Generalized q-sampling imaging; Machine learning; Resting-state functional magnetic resonance imaging |
| Boone M.; Roussel M.; Chauffert B.; Le Gars D.; Godefroy O. | Prevalence and profile of cognitive impairment in adult glioma: a sensitivity analysis | 2016 | Attention; Cognitive disorders; Executive disorders; Glioma; Neuropsychological tests |
| Wong S.S.; Case L.D.; Avis N.E.; Cummings T.L.; Cramer C.K.; Rapp S.R. | Cognitive functioning following brain irradiation as part of cancer treatment: Characterizing better cognitive performance | 2019 | brain tumor; cancer; cognition; fatigue; oncology; radiation; survivorship |
| Tymowski M.; Kaspera W.; Metta-Pieszka J.; Zarudzki Ł.; Ładziński P. | Neuropsychological assessment of patients undergoing surgery due to low-grade glioma involving the supplementary motor area | 2018 | Brain tumor; Glioma resection; Neuropsychological assesment; Supplementary motor area |
| Perrier J.; Viard A.; Levy C.; Morel N.; Allouache D.; Noal S.; Joly F.; Eustache F.; Giffard B. | Longitudinal investigation of cognitive deficits in breast cancer patients and their gray matter correlates: impact of education level | 2020 | Anxiety; Breast cancer; Cognition; Education level; Magnetic resonance imaging |
| Calvi E.; Marchetti M.; Santagata F.; Luppi C.; Coppo E.; Massaia M.; Isaia G.C. | Similar neurocognitive patterns in patients treated with lenalidomide: chemobrain effect? | 2019 | chemobrain effect; Cognitive decline; cognitive impairment; lenalidomide; neuropsychology |
| Kuśmierek M.; Jasionowska J.; Maruszewska P.; Kalinka-Warzocha E.; Gałecki P.; Mikołajczyk I.; Talarowska M. | The impact of cancer treatment on cognitive efficiency: Chemobrain – does it exist? | 2020 | Chemobrain; Chemotherapy; Cognition |
| Wang Q.; Qi F.; Song X.; Di J.; Zhang L.; Zhou Y.; Lu X.; Chang J.; Yu Y. | A prospective longitudinal evaluation of cognition and depression in postoperative patients with high-grade glioma following radiotherapy and chemotherapy | 2018 | Depression; high-grade glioma; mini-mental state examination; radiotherapy; temozolomide |

| | | | |
|---|---|---|---|
| Finke C.; Bartels F.; Lütt A.; Prüss H.; Harms L. | High prevalence of neuronal surface autoantibodies associated with cognitive deficits in cancer patients | 2017 | Autoantibodies; Cancer; Cognitive impairment; Dementia; NMDA receptor; Paraneoplastic syndrome |
| Pasqual E.; Boussin F.; Bazyka D.; Nordenskjold A.; Yamada M.; Ozasa K.; Pazzaglia S.; Roy L.; Thierry-Chef I.; de Vathaire F.; Benotmane M.A.; Cardis E. | Cognitive effects of low dose of ionizing radiation – Lessons learned and research gaps from epidemiological and biological studies | 2021 | Atomic bombing; Chernobyl accident; Cognition; Ionizing radiation; Low doses; Medical radiation; MELODI; Neurodegeneration; Neurodevelopment |
| Cordes J.; Woite M.; Engelke C.; Regenbrecht G.; Kahl K.G.; Schmidt-Kraepelin C.; Henning U.; Kamp D.; Klimke A. | Hormone replacement therapy with L-thyroxine promotes working memory and concentration in thyroidectomized female patients after differentiated thyroid carcinoma | 2020 | cognitive function; depression; hypothyroidism; levothyroxine therapy |
| Ponto L.L.B.; Menda Y.; Magnotta V.A.; Yamada T.H.; Denburg N.L.; Schultz S.K. | Frontal hypometabolism in elderly breast cancer survivors determined by [18F]fluorodeoxyglucose (FDG) positron emission tomography (PET): A pilot study | 2015 | brain metabolism; breast cancer; chemobrain; FDG; [<sup>18</sup>F]fluorodeoxyglucose |
| Su Y.; Pu Y.; Zhao Z.; Yang X. | Influence of combined epidural anesthesia on cognitive function, inflammation and stress response in elderly liver cancer patients undergoing surgery | 2020 | Cognitive function; Combined epidural anesthesia; Elderlyliver cancer patients undergoing surgery; Inflammation; Stress response |
| van Nieuwenhuizen D.; Douw L.; Klein M.; Peerdeman S.M.; Heimans J.J.; Reijneveld J.C.; Stam C.J.; Hillebrand A. | Cognitive functioning and functional brain networks in postoperative WHO grade I meningioma patients | 2018 | Cognitive functioning; Functional connectivity; Magnetoencephalography; Meningioma; Minimum spanning tree; Resting-state networks |
| Racine C.A.; Li J.; Molinaro A.M.; Butowski N.; Berger M.S. | Neurocognitive function in newly diagnosed low-grade glioma patients undergoing surgical resection with awake mapping techniques | 2015 | Awake mapping; Cognition; Low-grade glioma; Neuropsychology; Surgical resection |
| Bedard M.; Verma S.; Collins B.; Song X.; Paquet L. | Prospective memory impairment in chemotherapy-exposed early breast cancer survivors: Preliminary evidence from a clinical test | 2016 | breast cancer; chemobrain; chemotherapy; cognitive deficits; prospective memory impairment |

| | | | |
|---|---|---|---|
| Allen B.D.; Acharya M.M.; Lu C.; Giedzinski E.; Chmielewski N.N.; Quach D.; Hefferan M.; Johe K.K.; Limoli C.L. | Remediation of Radiation-Induced Cognitive Dysfunction through Oral Administration of the Neuroprotective Compound NSI-189 | 2018 | |
| Incekara F.; Satoer D.; Visch-Brink E.; Vincent A.; Smits M. | Changes in language white matter tract microarchitecture associated with cognitive deficits in patients with presumed low-grade glioma | 2019 | Cognition; Diffusion tensor imaging; Glioma; Oncology; White matter tracts |
| Hermelink K. | Chemotherapy and cognitive function in breast cancer patients: The so-called chemo brain | 2015 | |
| Li T.-Y.; Chen V.C.-H.; Yeh D.-C.; Huang S.-L.; Chen C.-N.; Chai J.-W.; Chen C.C.-C.; Weng J.-C. | Investigation of chemotherapy-induced brain structural alterations in breast cancer patients with generalized q-sampling MRI and graph theoretical analysis | 2018 | Breast cancer; Chemotherapy; Generalized q-sampling imaging; Graph theoretical analysis; Multiple regression analysis; Network-based statistical analysis; Voxel-based statistical analysis |
| Cochereau J.; Herbet G.; Duffau H. | Patients with incidental WHO grade II glioma frequently suffer from neuropsychological disturbances | 2016 | Cognitive disturbances; Incidental tumor; Neuropsychological assessment; Quality of life; WHO grade II glioma |
| Soto-Perez-de-Celis E.; Sun C.-L.; Tew W.P.; Mohile S.G.; Gajra A.; Klepin H.D.; Owusu C.; Gross C.P.; Muss H.B.; Lichtman S.M.; Chapman A.E.; Cohen H.J.; Dale W.; Kim H.; Fernandes S.; Katheria V.; Hurria A. | Association between patient-reported hearing and visual impairments and functional, psychological, and cognitive status among older adults with cancer | 2018 | activities of daily living; anxiety; cognitive dysfunction; depression; geriatric oncology; hearing loss; neoplasms; older adults; vision disorders |
| Maialetti A.; Maschio M.; Zarabla A.; Polimadei C.; Papa E.; Villani V.; Giannarelli D. | Multimodal pathway for brain tumor-related epilepsy patients: Observational study | 2020 | brain tumor-related epilepsy (BTRE); multimodal approach; quality of life; rehabilitation; social relations; supportive network |
| Maschio M.; Dinapoli L.; Fabi A.; Giannarelli D.; Cantelmi T. | Cognitive rehabilitation training in patients with brain tumor-related epilepsy and cognitive deficits: a pilot study | 2015 | Antiepileptic drugs; Brain tumor; Brain tumor-related epilepsy; Cognitive deficits; Cognitive rehabilitation |

| | | | |
|---|---|---|---|
| Vance D.E.; Frank J.S.; Bail J.; Triebel K.L.; Niccolai L.M.; Gerstenecker A.; Meneses K. | Interventions for cognitive deficits in breast cancer survivors treated with chemotherapy | 2017 | Breast cancer survivors; Chemotherapy; Cognition; Cognitive intervention; Cognitive remediation therapy |
| Kurita G.P.; Sandvad M.; Lundorff L.; De Mattos-Pimenta C.A.; Hojsted J.; Sjogren P. | Assessment of cognitive function in patients with metastatic cancer: Are we using the right tools? | 2018 | Cancer; Cognition; Cognitive screening; Neuropsychological assessment; Symptom validity |
| Feng L.R.; Espina A.; Saligan L.N. | Association of Fatigue Intensification with Cognitive Impairment during Radiation Therapy for Prostate Cancer | 2018 | Cancer-related fatigue; Cognitive impairment; Prostate cancer; Radiation therapy |
| Barbaret C.; Delgado-Guay M.O.; Sanchez S.; Brosse C.; Ruer M.; Rhondali W.; Monsarrat L.; Michaud P.; Schott A.M.; Bruera E.; Filbet M. | Inequalities in Financial Distress, Symptoms, and Quality of Life Among Patients with Advanced Cancer in France and the U.S. | 2019 | Advanced cancer; Financial distress; Palliative care; Quality of life; Symptom distress |
| Kam J.W.Y.; Boyd L.A.; Hsu C.L.; Liu-Ambrose T.; Handy T.C.; Lim H.J.; Hayden S.; Campbell K.L. | Altered neural activation during prepotent response inhibition in breast cancer survivors treated with chemotherapy: an fMRI study | 2016 | Breast cancer survivors; Chemotherapy; Cognitive deficits; Functional MRI; Prepotent response inhibition |
| Frank J.S.; Vance D.E.; Triebel K.L.; Meneses K.M. | Cognitive deficits in breast cancer survivors after chemotherapy and hormonal therapy | 2015 | breast cancer; chemobrain; chemotherapy; cognitive deficits; hormonal therapy; neuropsychological performance |
| Alexander J.F.; Seua A.V.; Arroyo L.D.; Ray P.R.; Wangzhou A.; Heiβ-Lückemann L.; Schedlowski M.; Price T.J.; Kavelaars A.; Heijnen C.J. | Nasal administration of mitochondria reverses chemotherapy-induced cognitive deficits | 2021 | Chemobrain; Mesenchymal stem cell; Mitochondria; Nasal delivery; Nrf2 |
| Giffard B.; Perrotin A.; Allain P.; Dayan J.; Eustache F.; Grellard J.-M.; Faveyrial A.; Joly F.; Lange M. | The role of metamemory on cognitive complaints in cancer patients | 2020 | cancer patients; cognition; memory; metacognition; metacognitive monitoring; metamemory; neuropsychology |

| | | | |
|---|---|---|---|
| Lu X.-Y.; Chen M.; Chen D.-H.; Li Y.; Liu P.-T.; Liu Y. | Remifentanil on T lymphocytes, cognitive function and inflammatory cytokines of patients undergoing radical surgery for cervical cancer | 2018 | Cervical cancer; Cognitive dysfunction; Fentanyl; Inflammatory cytokines; Remifentanil |
| Pravettoni G.; Dell'Osso B.; Bocci T.; Cortese F.; Ferrucci R.; Lampis V.; Rosci C.; Priori A. | Psychiatric, behavioral, and cognitive disorders in patients with extracranial cancers | 2018 | extracranial cancer; Key wordsCognitive impairment; neuropathology; psychiatric symptoms; treatment |
| Sharpley C.F.; Bitsika V.; Christie D.R.H. | Variability in Depressive Symptoms of Cognitive Deficit and Cognitive Bias During the First 2 Years After Diagnosis in Australian Men With Prostate Cancer | 2016 | depression; development and aging; maturation; mental health; prostate cancer |
| Kang H.-L.; Chen V.C.-H.; Hung W.-L.; Hsiao H.-P.; Wang W.-H. | Preliminary comparison of neuropsychological performance in patients with non-small-cell lung cancer treated with chemotherapy or targeted therapy | 2019 | Anxiety; Depression; Neuropsychological performance; Non-small-cell lung cancer; Psychomotor speed |
| Gehring K.; Taphoorn M.J.B.; Sitskoorn M.M.; Aaronson N.K. | Predictors of subjective versus objective cognitive functioning in patients with stable grades II and III glioma | 2015 | Cognitive function; Glioma; Mental health; Predictors; Self-reported cognitive symptoms |
| Smith A.E.; Slivicki R.A.; Hohmann A.G.; Crystal J.D. | The chemotherapeutic agent paclitaxel selectively impairs learning while sparing source memory and spatial memory | 2017 | Episodic memory; Learning; Paclitaxel; Rats; Source memory; Spatial memory |
| Barailler H.; Dousset L.; Mertens C.; Maurel A.; Gérard E.; Prey S.; Dutriaux C.; Beylot-Barry M.; Pham-Ledard A. | Impact on quality of life and autonomy of patients aged over 75 years treated with anti-PD-1 for metastatic melanoma: A single-centre prospective study; [Impact sur la qualité de vie et l'autonomie des patients de plus de 75 ans traités par anti-PD-1 pour un mélanome métastatique : étude prospective monocentrique] | 2020 | Anti-PD-1; Elderly subjects; Immunotherapy; Melanoma; Quality of life |
| Le Rhun E.; Delbeuck X.; Lefeuvre-Plesse C.; Kramar A.; Skrobala E.; Pasquier F.; Bonneterre J. | A phase III randomized multicenter trial evaluating cognition in post-menopausal breast cancer patients receiving adjuvant hormonotherapy | 2015 | Aromatase inhibitors; Breast cancer; Chemobrain; Cognitive disorders; Hormonotherapy; Tamoxifen |
| Meskal I.; Gehring K.; van der Linden S.D.; Rutten G.-J.M.; Sitskoorn M.M. | Cognitive improvement in meningioma patients after surgery: clinical relevance of computerized testing | 2015 | Brain tumor surgery; Cognitive functioning; Meningioma; Quality of life |

| | | | |
|---|---|---|---|
| Alirezaei Z.; Amouheidari A.; Hassanpour M.; Davanian F.; Iraji S.; Shokrani P.; Nazem-Zadeh M.-R. | Early Detection of Radiation-Induced Injury and Prediction of Cognitive Deficit by MRS Metabolites in Radiotherapy of Low-Grade Glioma | 2021 | |
| Korc-Grodzicki B.; Alici Y.; Nelson C.; Alexander K.; Manna R.; Gangai N.; Shen M.J.; Parker P.A.; Banerjee S.C. | Addressing the quality of communication with older cancer patients with cognitive deficits: Development of a communication skills training module | 2020 | Cognitive syndrome; Communication skills training; Geriatrics; Oncology; Standardized patient assessments |
| Kaiser J.; Dietrich J.; Amiri M.; Rüschel I.; Akbaba H.; Hantke N.; Fliessbach K.; Senf B.; Solbach C.; Bledowski C. | Cognitive Performance and Psychological Distress in Breast Cancer Patients at Disease Onset | 2019 | attention; breast cancer; cognitive functions; memory; psychological distress |
| Ma Q.; Zeng L.-L.; Qin J.; Luo Z.; Su J.; Wu D.; Qiu S.; Hu D. | Radiation-induced cerebellar-cerebral functional connectivity alterations in nasopharyngeal carcinoma patients | 2017 | Biomarker; cerebellum; cognitive deficit; functional connectivity; nasopharyngeal carcinoma; radiotherapy |
| Skeie B.S.; Eide G.E.; Flatebø M.; Heggdal J.I.; Larsen E.; Bragstad S.; Pedersen P.-H.; Enger P.Ø. | Quality of life is maintained using Gamma Knife radiosurgery: A prospective study of a brain metastases patient cohort | 2017 | Brain metastases; Functional Assessment of Cancer Therapy-Brain; Gamma knife radiosurgery; Oncology; Quality of life; Stereotactic radiosurgery |
| Rijnen S.J.M.; Meskal I.; Bakker M.; De Baene W.; Rutten G.-J.M.; Gehring K.; Sitskoorn M.M. | Cognitive outcomes in meningioma patients undergoing surgery: Individual changes over time and predictors of late cognitive functioning | 2019 | cognition; individual changes; meningioma; predictors; reliable change index |
| Lange M.; Heutte N.; Noal S.; Rigal O.; Kurtz J.-E.; Lévy C.; Allouache D.; Rieux C.; Lefel J.; Clarisse B.; Leconte A.; Veyret C.; Barthélémy P.; Longato N.; Tron L.; Castel H.; Eustache F.; Giffard B.; Joly F. | Cognitive Changes After Adjuvant Treatment in Older Adults with Early-Stage Breast Cancer | 2019 | Aging; Breast neoplasms; Chemotherapy; Cognition disorders; Cognitive changes |

| | | | |
|---|---|---|---|
| Piccirillo J.F.; Hardin F.M.; Nicklaus J.; Kallogjeri D.; Wilson M.; Ma C.X.; Coalson R.S.; Shimony J.; Schlaggar B.L. | Cognitive impairment after chemotherapy related to atypical network architecture for executive control | 2015 | Breast neoplasms; Chemotherapy; Cognitive disorders; Complications; Diagnostic imaging; Neuroimaging |
| Stessin A.M.; Banu M.A.; Clausi M.G.; Berry N.; Boockvar J.A.; Ryu S. | FTY720/fingolimod, an oral S1PR modulator, mitigates radiation induced cognitive deficits | 2017 | Brain tumor stem cells; Cognitive dysfunction; Neural stem/progenitor cells; Radiation mitigator; S1P pathway |
| van Deudekom F.J.; van der Velden L.-A.; Zijl W.H.; Schimberg A.S.; Langeveld A.P.; Slingerland M.; Blauw G.J.; Mooijaart S.P. | Geriatric assessment and 1-year mortality in older patients with cancer in the head and neck region: A cohort study | 2019 | cognitive disorders; geriatric oncology; head and neck cancer; physical functioning; social functioning |
| Van Dyk K.; Hunter A.M.; Ercoli L.; Petersen L.; Leuchter A.F.; Ganz P.A. | Evaluating cognitive complaints in breast cancer survivors with the FACT-Cog and quantitative electroencephalography | 2017 | Breast cancer; Cognition; Cognitive complaints; Quantitative electroencephalography; Self-report |
| Schiff D.; Lee E.Q.; Nayak L.; Norden A.D.; Reardon D.A.; Wen P.Y. | Medical management of brain tumors and the sequelae of treatment | 2015 | Bevacizumab; Brain tumor; Chemotherapy; Cognition; Complications; Corticosteroids; Fatigue; Mood; Seizure; Symptom management; Vasogenic edema; Venous thromboembolism |
| Capo G.; Skrap M.; Guarracin I.; Isola M.; Battistella C.; Ius T.; Tomasino B. | Cognitive functions in repeated glioma surgery | 2020 | Awake surgery; Diffuse low surgery; Neurocognition; Neuropsychological assessment |
| Overcash J.; Perry M. | Cognitive screening: Using the clock-drawing test to assess for preexisting deficits in older women diagnosed with breast cancer | 2017 | Clock-drawing test; Comprehensive geriatric assessment; Dementia; Geriatrics; Oncology |
| Small B.J.; Jim H.S.L.; Eisel S.L.; Jacobsen P.B.; Scott S.B. | Cognitive performance of breast cancer survivors in daily life: Role of fatigue and depressed mood | 2019 | breast cancer; cognition; depression; ecological momentary assessment; fatigue; memory; oncology; quality of life; survivorship |

| | | | |
|---|---|---|---|
| De Baene W.; Jansma M.J.; Schouwenaars I.T.; Rutten G.-J.M.; Sitskoorn M.M. | Task-evoked reconfiguration of the fronto-parietal network is associated with cognitive performance in brain tumor patients | 2020 | Brain tumor patients; Cognitive flexibility; Fronto-parietal network; Task-evoked network reconfiguration; Working memory |
| Karunamuni R.; Tringale K.R.; Burkeen J.; Tibbs M.D.; Huynh-Le M.-P.; Bahrami N.; Marshall D.; Seibert T.M.; McDonald C.R.; Hattangadi-Gluth J.A. | Multi-domain neurocognitive classification of primary brain tumor patients prior to radiotherapy on a prospective clinical trial | 2020 | Global deficit score; Neurocognitive function; Primary brain tumors; Radiotherapy |
| Van Arsdale A.; Rosenbaum D.; Kaur G.; Pinto P.; Kuo D.Y.-S.; Barrera R.; Goldberg G.L.; Nevadunsky N.S. | Prevalence and factors associated with cognitive deficit in women with gynecologic malignancies | 2016 | Anxiety; Cognition; Depression; Gynecologic malignancies; Pain; Quality of life |
| Wang C.; Zhao Y.; Wang L.; Pan S.; Liu Y.; Li S.; Wang D. | C-phycocyanin Mitigates Cognitive Impairment in Doxorubicin-Induced Chemobrain: Impact on Neuroinflammation, Oxidative Stress, and Brain Mitochondrial and Synaptic Alterations | 2021 | C-phycocyanin; Chemotherapy; Cognitive; Doxorubicin; Mitochondria |
| Jarzemski P.; Brzoszczyk B.; Popiołek A.; Stachowicz-Karpińska A.; Golota S.; Bieliński M.; Borkowska A. | Cognitive function, depression, and anxiety in patients undergoing radical prostatectomy with and without adjuvant treatment | 2019 | Anxiety; Cognition; Depression; Prostate cancer; Sexual dysfunction |
| Fjalldal S.; Follin C.; Svärd D.; Rylander L.; Gabery S.; Petersén A.; Van Westen D.; Sundgren P.C.; Björkman-Burtscher I.M.; Lätt J.; Ekman B.; Johanson A.; Erfurth E.M. | Microstructural white matter alterations and hippocampal volumes are associated with cognitive deficits in craniopharyngioma | 2018 | |
| Yao C.; Rich J.B.; Tirona K.; Bernstein L.J. | Intraindividual variability in reaction time before and after neoadjuvant chemotherapy in women diagnosed with breast cancer | 2017 | attention; breast cancer; chemotherapy; cognition; executive function; intraindividual variability |

| | | | |
|---|---|---|---|
| Rydelius A.; Lätt J.; Kinhult S.; Engelholm S.; Van Westen D.; Pihlsgård M.; Bengzon J.; Sundgren P.C.; Lilja Å. | Longitudinal study of cognitive function in glioma patients treated with modern radiotherapy techniques and standard chemotherapy | 2020 | |
| Simó M.; Rifà-Ros X.; Vaquero L.; Ripollés P.; Cayuela N.; Jové J.; Navarro A.; Cardenal F.; Bruna J.; Rodríguez-Fornells A. | Brain functional connectivity in lung cancer population: an exploratory study | 2018 | Chemotherapy; Default mode network; Functional connectivity; Lung cancer; Resting-state functional magnetic resonance imaging |
| Cayuela N.; Jaramillo-Jiménez E.; Càmara E.; Majós C.; Vidal N.; Lucas A.; Gil-Gil M.; Graus F.; Bruna J.; Simó M. | Cognitive and brain structural changes in long-term oligodendroglial tumor survivors | 2019 | cognition; long-term survivors; neurotoxicity; oligodendroglioma; radiotherapy |
| Abete Fornara G.; Di Cristofori A.; Bertani G.A.; Carrabba G.; Zarino B. | Constructional Apraxia in Older Patients with Brain Tumors: Considerations with an Up-To-Date Review of the Literature | 2018 | Constructional apraxia; Elderly glioma; Elderly meningioma; Neuropsychology; Neurosurgery |
| Ren W.-T.; Li Y.-X.; Wang K.; Gao L.; Yi J.-L.; Huang X.-D.; Luo J.-W.; Wu R.-Y.; Yang Y.; Wang J.-Y.; Wang W.-Q.; Wang J.-B.; Ye F.; Ouyang H.; Dai J.-R. | Cerebral functional abnormalities in patients with nasopharyngeal carcinoma after radiotherapy: An observational magnetic resonance resting-state study | 2019 | Functional; MRI; Nasopharyngeal carcinoma; Radiotherapy |
| Ribeiro M.; Durand T.; Roussel M.; Feuvret L.; Jacob J.; Psimaras D.; Noel G.; Keller A.; Bompaire F.; Hoang-Xuan K.; Bernier M.-O.; Godefroy O.; Ricard D. | Sensitivity of the Montreal Cognitive Assessment in screening for cognitive impairment in patients with newly diagnosed high-grade glioma | 2020 | Cognitive assessment; Cognitive deficits; Cognitive screening; High-grade glioma; Neuropsychology |
| Muto J.; Dezamis E.; Rigaux-Viode O.; Peeters S.; Roux A.; Zanello M.; Mellerio C.; | Functional-Based Resection Does Not Worsen Quality of Life in Patients with a Diffuse Low-Grade Glioma Involving Eloquent Brain Regions: A Prospective Cohort Study | 2018 | Ability to work; Cognitive functioning; Diffuse low-grade glioma; Intraoperative functional mapping; Surgery |

| | | | |
|---|---|---|---|
| Sauvageon X.; Varlet P.; Oppenheim C.; Pallud J. | | | |
| Lang S.; Gaxiola-Valdez I.; Opoku-Darko M.; Partlo L.A.; Goodyear B.G.; Kelly J.J.P.; Federico P. | Functional Connectivity in Frontoparietal Network: Indicator of Preoperative Cognitive Function and Cognitive Outcome Following Surgery in Patients with Glioma | 2017 | Cognition; Frontoparietal network; Neurooncology; Neurosurgery; Resting-state functional connectivity |
| Durand T.; Jacob S.; Lebouil L.; Douzane H.; Lestaevel P.; Rahimian A.; Psimaras D.; Feuvret L.; Leclercq D.; Brochet B.; Tamarat R.; Milliat F.; Benderitter M.; Vayatis N.; Noël G.; Hoang-Xuan K.; Delattre J.-Y.; Ricard D.; Bernier M.-O. | EpiBrainRad: An epidemiologic study of the neurotoxicity induced by radiotherapy in high grade glioma patients | 2015 | Cognitive impairments; Leukoencephalopathy; Neurotoxicity; Quality-of-life; Radiotherapy |
